# Two related families of metal transferases, ZNG1 and ZNG2, are involved in acclimation to poor Zn nutrition in Arabidopsis

**DOI:** 10.3389/fpls.2023.1237722

**Published:** 2023-10-25

**Authors:** Lifang Zhang, Janeen Braynen, Audrey Fahey, Kriti Chopra, Paolo Cifani, Dimiru Tadesse, Michael Regulski, Fangle Hu, Hubertus J. J. van Dam, Meng Xie, Doreen Ware, Crysten E. Blaby-Haas

**Affiliations:** ^1^ Cold Spring Harbor Laboratory, Cold Spring Harbor, NY, United States; ^2^ Computational Science Initiative, Brookhaven National Laboratory, Upton, NY, United States; ^3^ Biology Department, Brookhaven National Laboratory, Upton, NY, United States; ^4^ Condensed Matter Physics and Materials Science Department, Brookhaven National Laboratory, Upton, NY, United States; ^5^ USDA ARS NAA Robert W. Holley Center for Agriculture and Health, Agricultural Research Service, Ithaca, NY, United States; ^6^ Department of Energy Joint Genome Institute, Lawrence Berkeley National Laboratory, Berkeley, CA, United States; ^7^ Molecular Foundry, Lawrence Berkeley National Laboratory, Berkeley, CA, United States

**Keywords:** CobW, COG0523, MetAP1, metallochaperone, MAP1, NME

## Abstract

Metal homeostasis has evolved to tightly modulate the availability of metals within the cell, avoiding cytotoxic interactions due to excess and protein inactivity due to deficiency. Even in the presence of homeostatic processes, however, low bioavailability of these essential metal nutrients in soils can negatively impact crop health and yield. While research has largely focused on how plants assimilate metals, acclimation to metal-limited environments requires a suite of strategies that are not necessarily involved in metal transport across membranes. The identification of these mechanisms provides a new opportunity to improve metal-use efficiency and develop plant foodstuffs with increased concentrations of bioavailable metal nutrients. Here, we investigate the function of two distinct subfamilies of the nucleotide-dependent metallochaperones (NMCs), named ZNG1 and ZNG2, that are found in plants, using *Arabidopsis thaliana* as a reference organism. AtZNG1 (AT1G26520) is an ortholog of human and fungal ZNG1, and like its previously characterized eukaryotic relatives, localizes to the cytosol and physically interacts with methionine aminopeptidase type I (AtMAP1A). Analysis of At*ZNG1*, At*MAP1A*, At*MAP2A*, and At*MAP2B* transgenic mutants are consistent with the role of Arabidopsis ZNG1 as a Zn transferase for AtMAP1A, as previously described in yeast and zebrafish. Structural modeling reveals a flexible cysteine-rich loop that we hypothesize enables direct transfer of Zn from AtZNG1 to AtMAP1A during GTP hydrolysis. Based on proteomics and transcriptomics, loss of this ancient and conserved mechanism has pleiotropic consequences impacting the expression of hundreds of genes, including those involved in photosynthesis and vesicle transport. Members of the plant-specific family of NMCs, ZNG2A1 (AT1G80480) and ZNG2A2 (AT1G15730), are also required during Zn deficiency, but their target protein(s) remain to be discovered. RNA-seq analyses reveal wide-ranging impacts across the cell when the genes encoding these plastid-localized NMCs are disrupted.

## 1 Introduction

The prevalence of Zn in biology is thought to be tied to the relative non-toxic nature of this metal and its abundance in the environment (Williams, 2012; Eom and Song, 2019). As a protein cofactor, Zn is often found in hydrolytic enzymes, where it lends a chemical functionality that is not easily provided solely by amino acid side chains (Coleman, 1992; Andreini et al., 2006). Because Zn lacks the ability to perform redox chemistry, it is also commonly used to stabilize protein structures (Christianson, 1991; Krishna, 2003; Maret, 2005). Eukaryotes have taken advantage of the latter property leading to an expansion of Zn proteins, particularly Zn-finger transcription factors, in their proteomes (Dupont et al., 2010). In addition to serving essential roles in transcription, translation, and regulation of transcription and protein abundance, Zn availability is critical for chloroplast biology and photosynthesis (Randall and Bouma, 1973; OHKI, 1976; Shrotri et al., 1983; Henriques, 2001; Wang and Jin, 2005). For example, Zn is an essential cofactor for most classes of carbonic anhydrase (Moroney et al., 2001) and for proper RuBisCO folding (Wilson and Hayer-Hartl, 2018). The absence of Zn or substitution with the wrong metal ion often leads to inactivity of Zn-dependent proteins. For instance, substitution of Zn for other metal ions in methionine aminopeptidase type I (MetAP1), an essential ribosome-associated factor that cleaves the initiator methionine (iMet), leads to erroneous substrate specificity (Li et al., 2003). How MetAP1 and other Zn-dependent proteins acquire Zn to the exclusion of all other metals, especially when Zn is limiting, is poorly understood.

Plants have unique challenges to overcome to maintain Zn homeostasis. A sedentary lifestyle forces plants to make do with the nutrients they can assimilate from the soil around them. Zn-limiting environments can exist, such as those created by competition between organisms (Gielda and Diritaa, 2012; Chignell et al., 2018) or in low-bioavailable calcareous soils (Alloway, 2009). In the United States, up to 66% of cropland is considered Zn deplete (Fixen et al., 2005), while in many parts of the world, a poor-zinc plant-based diet is one of the prominent causes of hidden hunger (Bouis and Welch, 2010; Das and Padhani, 2022; Stangoulis and Knez, 2022). Plants have evolved strategies to ensure uptake and storage, both to stockpile Zn and sequester excess, but the fate of Zn ions after import into cells and before binding to Zn-dependent proteins is largely unknown. Given the large number of Zn-binding proteins, relative metal-binding affinities likely govern the ability of proteins to acquire Zn from labile, “easily” exchangeable, zinc complexes (Maret, 2009; Foster et al., 2014; Zlobin et al., 2019). However, plant genomes encode an ortholog of the recently described Zn chaperones (named ZNG1 for Zn-regulated GTPase metalloprotein activator 1) (Pasquini et al., 2022; Weiss et al., 2022), suggesting that plant intracellular Zn homeostasis may also involve a Zn-delivery protein.

ZNG1 is a eukaryote-specific subfamily of the nucleotide-dependent metallochaperones (NMCs) (Blaby-Haas and Merchant, 2023). These proteins are also sometimes referred to as cobalamin biosynthesis proteins because of similarity to the CobW protein from bacteria (Crouzet et al., 1991) or referred to as COG0523 (Haas et al., 2009), a phylogeny-based designation from the database of Clusters of Orthologous Groups of proteins (COGs) (Tatusov et al., 1997). The NMCs represent a large family that belongs to the G3E family of P-loop GTPases (G3E family) (Leipe et al., 2002; Haas et al., 2009). Members of the G3E family also include UreG and HypB involved in the incorporation of Ni into urease and Ni-Fe hydrogenase, respectively (Leipe et al., 2002; Vaccaro and Drennan, 2022). NMCs are distinguished from other G3E family members, such as UreG, HypB, and MeaB, by the presence of a metal-binding motif CxCC located between the Switch I and Walker B motifs in the GTPase domain that is represented by IPR012824 from the InterPro database and, often, a C-terminal domain of unknown function, which is represented by IPR011629. The best characterized NMCs to date are ZNG1 from *Saccharomyces cerevisiae* (Pasquini et al., 2022) and *Danio rerio* (Weiss et al., 2022), where *in vivo* and *in vitro* data suggest that Zn is transferred to the target metalloprotein MetAP1 in a GTP-dependent manner. The best support for direct transfer of Zn has been provided by work with ScZng1 from *S. cerevisiae*. Based on immunodetection of a MetAP1 substrate, *in vivo* ScMap1 activity is negatively impacted by both loss of Sc*ZNG1* and Zn limitation (Pasquini et al., 2022). *In vitro*, EDTA does not inhibit the activity of ScMap1 when Zn is provisioned pre-bound to ScZng1, but EDTA is inhibitory with free Zn (Pasquini et al., 2022). Further, the predicted structure of the ScZng1-ScMap1 complex supports direct transfer of Zn from the CxCC motif of ScZng1to the ScMap1 Zn-binding site (Pasquini et al., 2022). How GTP hydrolysis enables Zn transfer from ZNG1 proteins to their MetAP1 targets is unknown.

Given the conservation of biochemical function and involvement in the response to Zn limitation across yeast and animals, and presence of an ortholog in plants, we reasoned that ZNG1 function may also be conserved in plants. In this study, using *Arabidopsis thaliana* as a genetic model, we tested the impact of At*ZNG1* (AT1G26520) loss on growth, the transcriptome, and proteome during Zn deficiency. Analysis of *zng1* mutant growth during Zn deficiency is consistent with a conserved role of AtZNG1 in AtMAP1A function. We also observe that At*MAP1A* is important for growth during Zn deficiency, providing a rationale for maintaining *ZNG1* in plants. We also identified two plant-specific ZNG1 homologs from a separate and distinct NMC subfamily, which are involved in acclimation to Zn deficiency based on loss-of-function phenotypes. These plastid-localized transferases are hypothesized to deliver Zn to a yet-to-be-discovered Zn-dependent protein(s) in the plastid. These results present a new understanding of subcellular Zn homeostasis in plants and present new clues for resolving the role and discovering the target proteins of the putative Zn transferases in plastids.

## 2 Materials and methods

### 2.1 Sequence analyses

To identify NMCs, publicly available proteins in the Phytozome (Goodstein et al., 2012) and UniProt (Bateman et al., 2021) databases were searched for keyword matches to the hidden Markov model (HMM) corresponding to the CobW/HypB/UreG, nucleotide-binding domain (PF02492). This search against the Phytozome database resulted in the identification of 6 proteins encoded in the *A. thaliana* genome (*Arabidopsis thaliana* TAIR10): AT1G26520, annotated as “Cobalamin biosynthesis CobW-like protein”, AT1G80480, annotated as “plastid transcriptionally active 17”, AT1G15730, annotated as “Cobalamin biosynthesis CobW-like protein”, AT1G15310, annotated as “signal recognition particle 54 kDa subunit”, AT1G48900, annotated as “Signal recognition particle, SRP54 subunit protein”, and AT2G34470, annotated as “urease accessory protein”. AT1G15310 and AT1G48900 belong to the signal-recognition-associated GTPase family and are not annotated as containing the CobW/HypB/UreG, nucleotide-binding domain (PF02492) in the UniProt database. This discrepancy between databases is likely due to different parameters/thresholds used to detect domains. AT2G34470 is an experimentally characterized accessory factor for urease biosynthesis in *A. thaliana* (Witte et al., 2005). AT1G26520, AT1G80480, and AT1G15730 protein sequences contain the characteristic GCXCC motif between the Walker A and Walker B motifs of the GTPase domain and a “Cobalamin (vitamin B_12_) biosynthesis CobW-like, C-terminal (IPR011629)” domain that are indicative of the NMCs (Haas et al., 2009; Edmonds et al., 2021).

To select sequences for phylogenetic reconstruction, the resulting proteins from the Phytozome and UniProt searches were used to build a sequence similarity network (SSN) with the EFI-EST webtool (http://efi.igb.illinois.edu/efi-est/) (Gerlt et al., 2015); to reduce compute time, the UniRef90 representative sequences corresponding to the UniProt set were used. An alignment score of 75 was used to generate edges, and nodes were collapsed based on 55% sequence identity. The network was visualized with the Prefuse Force Directed Open CL layout in Cytoscape v3.10.0 (Shannon et al., 2003). For the SSN shown in the main text, clusters containing proteins with matches to IPR004400 (UreG) and IPR004392 (HypB) were removed, as were clusters containing three or fewer nodes. The node table is available as **Table S1**. The original network before removal of UreG and HypB is shown in **Figure S1**.

To construct the ZNG cluster tree, sequences in the protein cluster containing AT1G26520, AT1G80480, and AT1G15730 were extracted. AT2G34470 (UreG) was included as an outgroup. To ensure that the approach does not create a sampling bias, we also performed a BLASTp search against the UniProt database using AT1G26520, AT1G80480, and AT1G15730 as queries. For each query, 1000 protein sequence hits were collected and found to be already present in the extracted “ZNG” SSN cluster. The multiple sequence alignment was generated using MAFFT (Katoh and Standley, 2013) on the CIPRES Science Gateway (Miller et al., 2010) with default parameters. Poorly aligned sequences (missing key sequence motifs (CxCC or GTPase motifs) and/or truncated sequences likely representing inaccurate gene models) were removed, only the portion of the alignment corresponding to the highly conserved GTPase domain was retained, and columns with less than 50% occupancy were removed. The edited multiple sequence alignment is available in **Table S2**. The resulting ZNG tree containing 1,686 sequences was constructed using FastTreeMP on XSEDE (Price et al., 2010) with default parameters (JTT+CAT substitution model and 1000 bootstraps for a Shimodaira-Hasegawa test). The consensus trees were visualized and annotated with iTOL (Letunic and Bork, 2021); branches with less than 0.5 bootstrap support were deleted. The leaf information and consensus tree are available in Newick format in **Table S2**. Because the nodes in the SSN represent one or more sequences (that share 55% sequence identity or more), each leaf in the phylogenetic tree represents one or more sequences. The represented sequences can be found in **Table S2**.

The plant ZNG1 and ZNG2 phylogenetic trees were generated by aligning orthologs of AtZNG1 or AtZNG2A1/AtZNG2A2 encoded by selected plant genomes housed in the Phytozome database. The multiple sequence alignment was generated with COBALT (Papadopoulos and Agarwala, 2007) and can be found in **Table S3**. The IQ-TREE webtool was used for reconstruction of phylogenetic relationships under maximum likelihood (Nguyen et al., 2015; Trifinopoulos et al., 2016) using the JTT+G4 (Jones-Taylor-Thornton) as the best-fit substitution model according to Bayesian information criterion (BIC) scores and weights. The consensus trees were visualized and annotated with iTOL (Letunic and Bork, 2021); branches with less than 50 bootstrap support were deleted. The leaf information and consensus tree are available in Newick format in **Table S3**.

The alignment in **Figure S2** was generated with MUSCLE (Robert and Gouet, 2014; Madeira et al., 2022) and visualized with EsPript 3.0 https://espript.ibcp.fr (Robert and Gouet, 2014). Putative transit peptides shown in **Figure S2** were predicted with WoLF PSORT (Horton et al., 2007). Transit peptides for the *zng2a1 zng2a2* transcriptomics analysis were collected from SUBA5 (Hooper et al., 2017).

### 2.2 Structural modeling

Protein structures of the ZNG1 and ZNG2 proteins (AtZNG1: AT1G26520, AtZNG2A1: AT1G80480, and AtZNG2A2: AT1G15730) and methionine aminopeptidases (AtMAP1A: AT2G45240, AtMAP1B: AT3G25740, AtMAP1C: AT1G13270, AtMAP1D: AT4G37040) were predicted employing Alphafold2 (Jumper et al., 2021). Protein complexes were predicted using the multimer module of Alphafold2 (Evans et al., 2022) using only evolutionary information and without including any known homologous templates. The quality of the structures obtained were assessed through the residue level pLDDT scores (Jumper et al., 2021). The structure models generated were visualized and analyzed for identifying residue-level interactions using ChimeraX (Goddard et al., 2018) and PyMOL (The PyMOL Molecular Graphics System, Version 2.0 Schrödinger, LLC).

### 2.3 Plant material and growth conditions

The list of mutants used and generated in this study are available in **Tables 1**, **2**. T-DNA insertion lines in the *Arabidopsis thaliana* Columbia-0 (referred to throughout as Col-0) background were obtained from the Arabidopsis Biological Resource Center (ABRC). They were genotyped using the KAPA 3G Plant PCR Kit (Roche) with leaf discs as templates and sequenced to confirm the insertion sites using Sanger sequencing. The location of sequencing-verified T-DNA sites are shown in **Figure S3**. The locations of T-DNA inserts in SAIL_598_G04, SALK_002480C, and SALK_037227C are misannotated in TAIR; the correct locations are shown in **Figure S3**. Primers used are listed in **Table S4**. The *zng1* and *zng2a1 zng2a2* mutants were also analyzed with RNA-seq data to confirm impact of the mutations on gene expression (**Figures S4**, **S5**). The double and triple mutants were generated by crossing the corresponding single or double mutants and validating with PCR in the F2 generations. For each set of Col-0 and mutant growth comparisons, plants were bulked in the same batch before being used for phenotyping. For growth on solid media, seeds were surface sterilized with 20% bleach and dH_2_O and stratified in a cold room (4°C) for three days before sowing to half-strength MS medium (Murashige et al., 1962) containing macro- and micro-nutrients with or without added Zn. Either agarose or Phytagel was used as the gelling agent (indicated in figure legends and other sections of the Methods) for growth on plates. The medium was made using a commercially available macronutrient solution (Sigma M0654) supplemented with micronutrients with or without 4.3 mg/L zinc sulfate (ZnSO_4_•7H_2_O). The final components for the half-strength MS per liter: 0.5 g 2-(N-morpholino)ethanesulfonic acid (MES), 10 g sucrose, 825 mg ammonium nitrate (NH_4_NO_3_), 3.2 mg boric acid (H_3_BO_3_), 166 mg calcium chloride anhydrous (CaCl_2_), 0.01 mg cobalt chloride (CoCl_2_•6H_2_O), 0.01 mg cupric sulfate (CuSO_4_•5H_2_O), 18.63 mg Na_2_-EDTA, 13.9 mg ferrous sulfate (FeSO_4_•7H_2_O), 90.3 mg magnesium sulfate (MgSO_4_), 8.45 mg manganese sulfate (MnSO_4_•H_2_O), 0.5mg molybdic acid (Na_2_MoO_4_•2H_2_O), 0.42 mg potassium iodide (KI), 950 mg potassium nitrate (KNO_3_), and 85 mg potassium phosphate monobasic (KH_2_PO_4_). Th pH was adjusted to 5.7 with KOH. Although Zn is excluded in the dropout medium, referred throughout as “minus Zn”, the medium is not Zn-free, which is often a contaminant of gelling agents, salts, water, and culture vessels (Sinclair and Krämer, 2020). Because of the presence of EDTA, the speciation of trace metals is expected to differ between the medium with and without supplemented Zn. Nevertheless, RNA-seq analysis of metal-responsive genes reveals that the change in speciation results in the Zn-deficiency response but does not equate to induction of the Fe-, Cu-, or Mn-deficiency responses (**Figure S6**). All glassware were washed with HCl and dH_2_O. Plants were grown on plates or in soil (seeds treated the same as for growth on plates, but the seeds were stratified on soil) in a Percival growth chamber at 23°C of a 16 h-light/8 h dark cycle. Unless stated otherwise, the primary root length of seedlings from three plates with three seedlings each for each genotype and growth conditions were averaged; error bars represent the standard deviations and indicated P values were determined with a Student’s T-test.

**Table 1:** List of single mutants used in this study.

Locus ID	Name	Mutant name	T-DNA line	ABRC stock number	notes
AT1G26520	ZNG1	*zng1-2*	SALK_203432C	SALK_203432C	**Figure S5**
AT1G26520	ZNG1	*zng1-1*	SAIL_153_E10	CS807382	main figures, proteomics (roots), RNAseq (roots)
AT1G15730	ZNG2A1	*zng2a1-2*	SALK_037227C	SALK_037227C	main figures, RNAseq (shoots)
AT1G15730	ZNG2A1	*zng2a1-1*	SALK_002480C	SALK_002480C	
AT1G80480	ZNG2A2	*zng2a2-1*	SAIL_640_D09	CS863183	
AT1G80480	ZNG2A2	*zng2a2-2*	SALK_144276C	SALK_144276C	main figures, RNAseq (shoots)
AT2G45240	MAP1A	*map1a-1*	SALK_021985C	SALK_021985C	main figures
AT2G45240	MAP1A	*map1a-2*	SALK_097303C	SALK_097303C	**Figure S5**
AT3G25740	MAP1B	*map1b*	SALK_004655	SALK_004655	
AT1G13270	MAP1C	*map1c*	SALK_108786	CS65697	
AT4G37040	MAP1D	*map1d*	WISCDSLOX393-396J14	CS864411	
AT2G44180	MAP2A	*map2a*	GK-290A03	CS427747	
AT3G59990	MAP2B	*map2b*	SAIL_598_G04	CS825517	

**Table 2:** List of double and triple mutants generated in this study.

Locus ID	Mutant Name	Mother	Father	Notes
*AT3G25740 AT1G13270*	*map1b map1c*	SALK_004655	SALK_108786	not viable
*AT1G13270 AT3G25740*	*map1c map1b*	SALK_108786	SALK_004655	not viable
*AT1G13270 AT4G37040*	*map1c map1d*	SALK_108786	WiscDsLox393	not viable
*AT4G37040 AT1G13270*	*map1d map1c*	WiscDsLox393	SALK_108786	not viable
*AT3G25740 AT4G37040*	*map1b map1d*	SALK_004655	WiscDsLox393	
*AT4G37040 AT3G25740*	*map1d map1b*	WiscDsLox393	SALK_004655	
*AT2G44180 AT3G59990*	*map2a map2b*	GK-290A03	SAIL_598_G04	
*AT1G26520 AT2G44180 AT3G59990*	*zng1-1 map2a map2b*	SAIL_153_E10	GK-290A03 SAIL_598_G04	not viable
*AT2G44180 AT3G59990 AT1G26520*	*map2a map2b zng1-1*	GK-290A03 SAIL_598_G04	SAIL_153_E10	not viable
*AT1G26520 AT2G44180 AT3G59990*	*zng1-2 map2a map2b*	SALK_203432C	GK-290A03 SAIL_598_G04	not viable
*AT2G44180 AT3G59990 AT1G26520*	*map2a map2b zng1-2*	GK-290A03 SAIL_598_G04	SALK_203432C	not viable
*AT1G15730 AT1G80480*	*zng2a1 zng2a2*	SALK_037227C	SALK_144276C	
*AT1G80480 AT1G15730*	*zng2a2 zng2a1*	SALK_144276C	SALK_037227C	
*AT1G15730 AT1G80480 AT3G25740*	*zng2a1 zng2a2 map1b*	*SALK_037227C SALK_144276C*	SALK_004655	
*AT1G15730 AT1G80480 AT1G13270*	*zng2a1 zng2a2 map1c*	*SALK_037227C SALK_144276C*	SALK_108786	not viable
*AT1G15730 AT1G80480* AT4G37040	*zng2a1 zng2a2 map1d*	*SALK_037227C SALK_144276C*	WiscDsLox393	
AT1G26520 AT2G44180	*zng1-2 map2a*	SALK_203432C	GK-290A03	not viable
AT2G44180 AT1G26520	*map2a zng1-2*	GK-290A03	SALK_203432C	viable

### 2.4 Protein localization and BiFC assays

The subcellular localizations were tested in *A. thaliana* mesophyll protoplasts as previously described (Xie et al., 2020). The cDNAs were cloned into the transient expression vector pUC-pGWB505 using the gateway cloning system (Invitrogen) for C-terminal YFP fusions (Xie et al., 2018). A total of 10 µg of the pUC-pGWB505 construct was transfected into 100 µl of protoplasts (~2 x 10^4^ cells) to express tagged proteins. A set of previously published vectors was used for bimolecular fluorescence complementation (BiFC) (Lee et al., 2008). The cDNA of At*ZNG1* was cloned into the pSAT1-cCFP-C vector. The cDNA of At*MAP1A* was cloned into the pSAT1-nVenus-C vector. The expression cassettes of these constructs were then cloned into the transient expression vector pUC119-RCS for the BiFC test in *A. thaliana* mesophyll protoplasts. A total of 5 µg of each plasmid were co-transfected in 100 µl of protoplasts. After 16 h incubation under weak light at room temperature, protoplasts were collected and resuspended in cold W5 solution (Medgyesy et al., 1980) (2 mM MES pH 5.7, 154 mM NaCl, 125 mM CaCl_2_, and 5 mM KCl) before imaging by microscopy. Images were collected using a Leica TCS SP5 confocal microscope, equipped with 488 nm laser lines. The emission bandwidth for YFP and chloroplast autofluorescence was 500-530 nm and 600-680 nm, respectively. Images were processed using LAS X software (Leica).

### 2.5 Yeast-two-hybrid assays

To test the predicted interaction between AtZNG1 and AtMAP1A and to test whether the plastid-localized AtZNG2A1 and AtZNG2A2 proteins could interact with the plastid-localized methionine aminopeptidases, yeast-two-hybrid (Y2H) assays were performed using ProQuest Two-Hybrid Systems (ThermoFisher Scientific), which uses three different reporter genes (*HIS3, URA3*, and *lacZ*). The *HIS3* reporter gene provides a reporter for bait-prey interaction and enables positive selection (presence allows growth on medium lacking histidine); HIS3 can be inhibited in a dose-dependent manner by 3-Amino-1,2,4-Triazole (3AT). Growth in the presence of 3AT provides confidence that the bait-prey interaction is strong (because expression of HIS3 is high enough to overcome inhibition). The URA3 provides negative selection (transformants expressing URA3 cannot grow in the presence of 5-fluoroorotic acid (5FOA)). The *lacZ* gene provides an additional reporter for bait-prey interaction and results in a blue color when assayed with X-gal (5-bromo-4-chloro-3-indolyl-β-D-galactopyranoside). Full-length cDNAs were cloned into both pDEST22 and pDEST32 vectors and screens were carried out in reciprocal combinations as prey and bait for each pair of genes (**Table S5**), according to manufacturer’s instructions.

### 2.6 Preparation of plant materials for RNA-seq and proteomics

Seedlings of Col-0, *zng1*, and *zng2a1 zng2a2* were grown vertically on half-strength MS medium with or without Zn added to the micronutrient solution (as described in section 2.3), using Phytagel (Sigma P8169) as the gelling agent. Roots and shoots were collected 10 days after sowing (DAS). Three biological replicates were grown and collected: three biological replicates from roots (for the Col-0 and *zng1* RNA-seq and proteomics comparisons) or shoots (for the Col-0 and *zng2a1 zng2a2* RNA-seq comparisons). Biological replicates were grown and collected on different days (different batches), but control and mutants were grown on the same day, *e.g.*, replicate one samples for Col-0 and *zng1*, in plus or minus Zn, were grown and collected on the same day. All plates were rotated daily from back to front to keep the same light density and top and bottom to avoid subtle temperature differences inside the growth chamber. All tissues were harvested 10 DAS. Since they were grown vertically, roots and shoots were harvested separately. A Zymo Research RNA mini purification kit was used to isolate total RNA for shoots and roots according to the manufacturer’s protocol with slight modifications.

### 2.7 RNA-seq library construction and analysis

The quantity and quality of the extracted RNAs were assessed by Qubit (Thermo Fisher) and Bioanalyzer (Agilent). The preparation of sequencing libraries and sequencing was conducted by Azenta Life Sciences (South Plains, New Jersey). The libraries were subsequently sequenced using the Illumina HiSeq 2000 platform (Illumina, California, USA). The raw data images were transformed into sequencing information by base calling and stored as FastQ format files. The RNA-seq data in FastQ format have been deposited in the NCBI Sequences Read Archives (SRA) with accession number PRJNA984600.

Paired-end reads were aligned to the Arabidopsis TAIR10 genome using the STAR (v2.7.9) pipeline (Dobin and Gingeras, 2015). Trimmomatic (2013) was utilized for adapter filtering (Bolger et al., 2014). The reads were then assembled into transcripts using StringTie (v2.1.4) and downstream annotation employed the TAIR10 genome GTF annotation file obtained from (www.arabidopsis.org). Count matrices were generated from the StringTie GTF files to analyze differentially expressed genes and subsequently utilized in downstream analysis. Differential gene expression analysis was performed using DESeq2 (v1.34.0; (Anders and Huber, 2010)). A multiple-test corrected p-value and q-value were employed using the Benjamini and Hochberg method with a threshold of 0.05, along with a Log2foldchange criterion of >1 for up-regulated genes and <-1 for down-regulated genes (Benjamini and Hochberg, 1995). Functional analysis was conducted separately for less-abundant and more-abundant transcripts in each pairwise comparison. Gene Ontology (GO) analysis of all groups was carried out using AgriGO and BinGO, with the default parameters and a multiple test correction of 0.05 (Benjamini and Hochberg, 1995; Maere et al., 2005; Tian et al., 2017). Additionally, KEGG pathway enrichment analysis was performed using the clusterProfiler package in R. The heatmap was generated with Clustergrammer using z-score normalized TPM values and default parameters (Fernandez et al., 2017).

### 2.8 Preparation and mass spectrometry-based analysis of samples for TMT-labelled proteomics

In this study, we employed Tandem Mass Tags (TMT) coupled with mass spectrometry to perform a quantitative proteomic analysis. The intensity of reporter ions, produced during peptide fragmentation, servs as the metric for relative peptide abundance across multiple samples. To process samples for analysis, approximately 1 g of each sample (collected as described in section 2.6) was thawed on ice, dissolved in 500 μl 200 mM tetraethylammonium bromide (TEAB)/2% sodium dodecyl sulfate (SDS) and lysed using a probe sonicator. Lysates were cleared by centrifugation, and protein content was quantified using the bicinchoninic acid assay (BCA, Pierce). After quantification, 50 μg of each sample was loaded on a sodium dodecyl-sulfate polyacrylamide gel electrophoresis (SDS-PAGE) 10% gel (BioRad), and electrophoresis was performed applying 100 V for approximately 10 minutes (i.e., to let the sample enter the gel, without resolving it). Proteins were stained using GelCode Coomassie (BioRad), bands were excised, and destained incubating trice with 25% acetonitrile/100 mM TEAB. Cysteine residues were reduced with 3 mM Tris (2-carboxyethyl) phosphine (TCEP) for 30 min at 55°C, and alkylated using 10 mM 2-carboxyethyl methanethiosulfonate (CEMTS, Santa Cruz). Excess alkylating agent was removed washing trice with 100 mM TEAB, and 1 μg Sequencing Grade Modified Porcine Trypsin (Promega) was added to each sample for in-gel digestion (1:50 w/w protease:sample). Proteolysis was performed overnight at 37°C. Peptides were eluted in 200 mM TEAB/80% acetonitrile, and each sample was encoded with 1 mg TMT16-plex reagents (Thermo Scientific) for 90 minutes at room temperature under constant agitation. The labeling reaction was quenched with 5% hydroxylamine, and 50% of each reaction volume was pooled. The peptide pool was lyophilized by vacuum centrifugation, and peptides were fractionated by high-pH reversed phase chromatography in spin column format (Pierce). Fractions were lyophilized, and peptide pellets were resuspended in 20 μl 0.1% formic acid/5% dimethyl sulfoxide (DMSO by sonication.

For analysis by liquid chromatography-mass spectrometry (LC/MS), 1 μl of each peptide fraction was loaded on a 25 cm x 75 μm internal diameter (ID) column packed with Reprosil 1.9 μm C18 silica particles, and resolved on a 90 minutes 5-35% acetonitrile gradient in water (0.1% formate) at 200 nl/min. Gradient was delivered using an easyLC 1200 nano-chromatograph (Thermo Scientific) in line with the electrospray source. Eluting peptides were ionized by electrospray using a 10 μm tip ID silica emitter (Fossil Ion Tech) maintained at 2200 volts (V) compared to the mass spectrometer inlet, and transferred into a Lumos mass spectrometer (Thermo). The mass spectrometer (MS) was set to collect precursor scans at 120,000 resolution (m/z 380-2000 Th, AGC 100,000 ions, max injection time 50 ms) every 3 s. Using the data-dependent routine with dynamic exclusion (10 ppm tolerance, 30 s exclusion). Precursor ions were selected using the quadrupole (1.2 thomson (Th) isolation window) for fragmentation. Fragmentation was performed by higher-energy C-trap dissociation (HCD) at stepped 28,33,38% normalized, and centroided MS2 spectra were collected in the orbitrap at 60,000 resolution, with the first mass locked to 100 Th (automatic gain control (AGC) 200%, max injection time 118 ms). MS parameters were preliminary optimized in a pilot experiment to maximize coverage and dynamic range of quantification. Files were searched using the Mascot scoring function (Perkins et al., 1999) within ProteomeDiscoverer v.2.4 (Thermo), with mass tolerance set at 5 ppm for MS1, and 0.1 Da for MS2. Spectra were matched against the UniProt *A. thaliana* database (as of December 21, 2022), plus a database of common contaminants (cRAP). M-oxidation and N/Q-deamidation were set as variable modifications. Cysteine modification with CEMTS was set as fixed modification. Peptide-spectral matches were filtered to maintain a false-discovery rate (FDR) <1% using Percolator. Intensity of reporter ions (10 ppm integration window) was used as a quantitative metric for protein relative quantification, and fold-change was calculated by dividing the average reporter intensity for one sample divided by the average of another as indicated in text and figure legends. Only proteins identified by more than one peptide-spectral match were used for downstream analysis. Out of the 409,719 recorded MS2 spectra, 18.3% were successfully matched to TMT-labeled peptides. After excluding contaminants and proteins with insufficient quantitative or identification confidence, a finalized set of 3,838 proteins was subjected to subsequent analysis that included the use of a Student’s T-test to determine statistically significant differences (P value <0.05) between comparators (three biological replicates per genotype and growth condition). Data, statistics, averages, standard deviations, fold changes, and P values are available in **Table S6**. R packages pheatmap and ggplot2 were used for functional analysis and heat maps. The mass spectrometry proteomics data have been deposited to the ProteomeXchange Consortium via the PRIDE (Perez-Riverol et al., 2022) partner repository with the dataset identifier PXD042911.

## 3 Results

### 3.1 Land plant genomes encode two distinct families related to ZNG1

**Figure 1:**
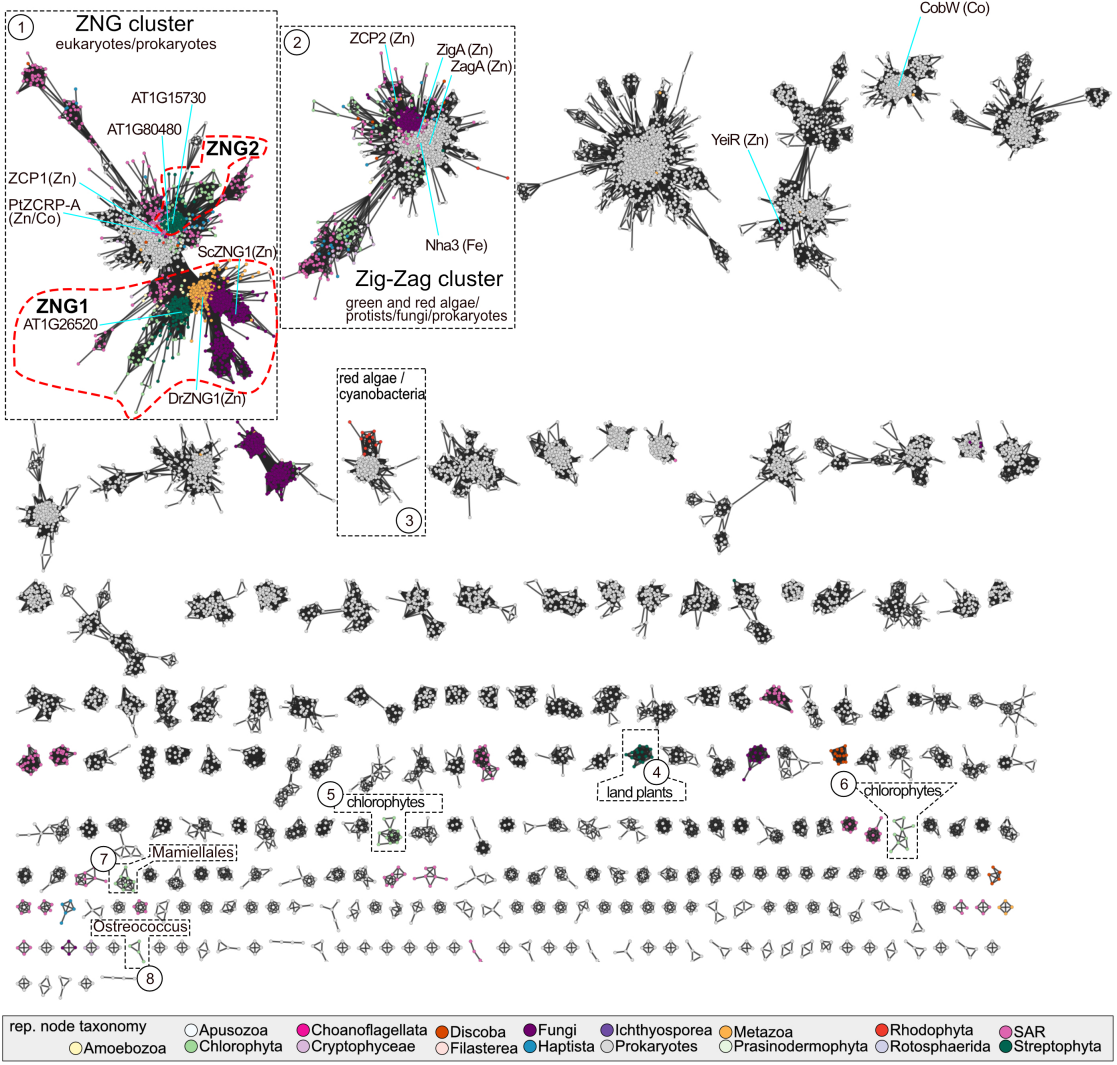
Sequence similarity network of the NMC family. Each node may represent one or more protein sequences and are colored based on the taxonomy of the representative sequence, according to the color key. Clusters containing members from Archaeplastida are numbered and highlighted with a black dotted line. The ZNG1 and ZNG2 subfamilies are outlined with a red dotted line. The major taxonomic lineages associated with each highlighted cluster are given. Nodes representing experimentally characterized family members are labeled with the protein name and known/putative metal that the activase has been connected to experimentally. SAR is an abbreviation for Stramenopiles, Alveolates, and Rhizaria. A table with node information in available in **Table S1**.

**Figure 2:**
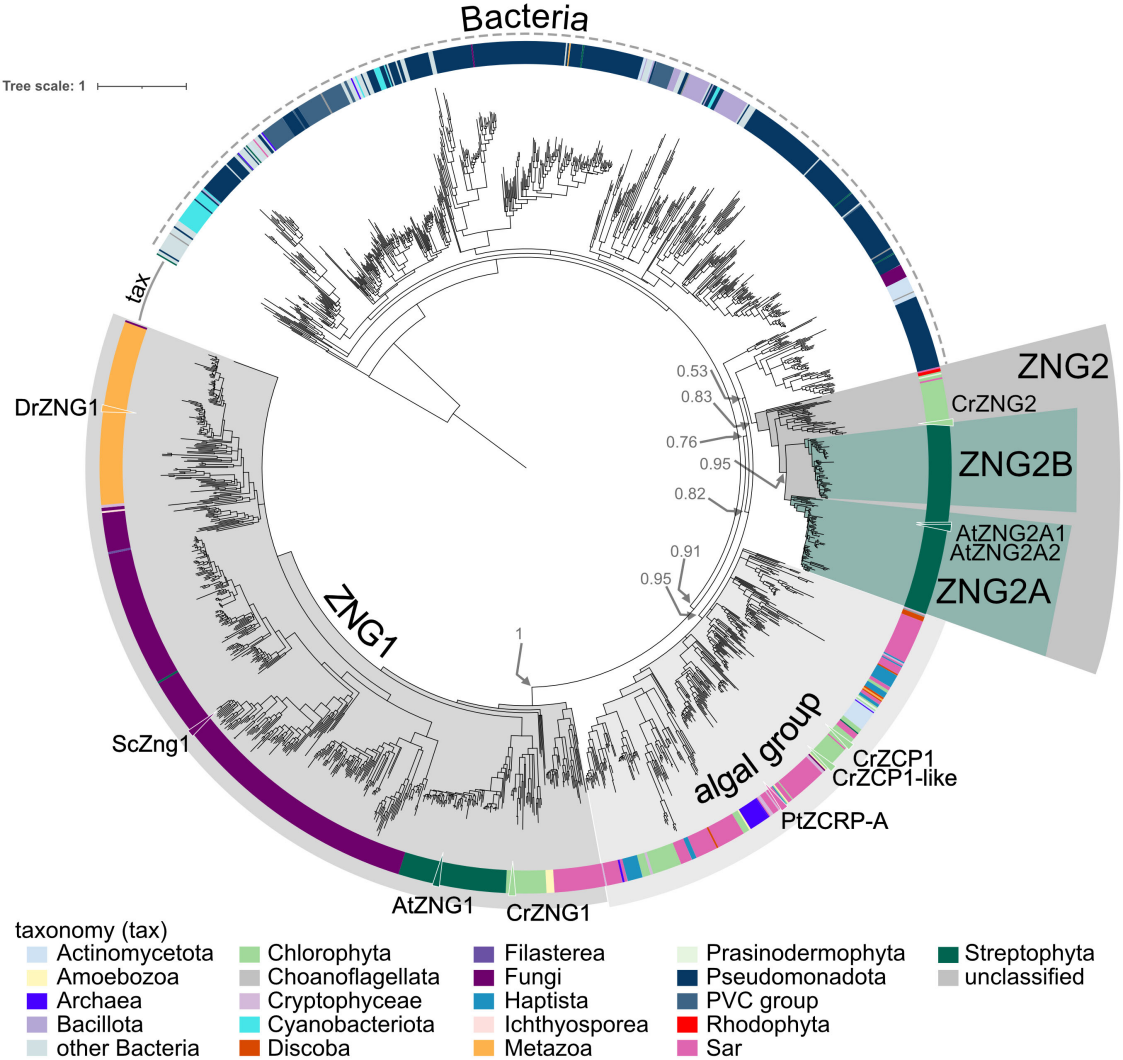
Approximately-maximum-likelihood phylogenetic tree of proteins from the ZNG cluster. The outer ring indicates taxonomic classification of each leaf according to the color key. Clades containing the ZNG1 and ZNG2 subfamilies, ZNG2A and ZNG2B subfamilies, and the ZCP1/ZCRP-A-like algal clade are labeled. Leaves representing ZNG1 orthologs from *Arabidopsis thaliana* (AtZNG1), *Chlamydomonas reinhardtii* (CrZNG1), *Saccharomyces cerevisiae* (ScZng1), and *Danio rerio* (DrZNG1), ZNG2 proteins from *A*. *thaliana* (AtZNG2A1 and AtZNG2A2), *C. reinhardtii* (CrZNG2), and the closely related ZNG-like proteins from the diatom *Phaeodactylum tricornutum* (PtZCRP-A) and *C. reinhardtii* (CrZCP1 and CrZCP1-like) are labeled with a triangle and protein name. Associated data and leaf information is given in **Table S2**.

A sequence similarity network (SSN) of the NMC proteins revealed three distinct protein clusters that contain members encoded by land plant genomes (**Figure 1**). Plant proteins in the largest cluster “ZNG” can be divided into two subclusters. The ZNG1 subcluster contains previously characterized ZNG1 proteins from yeast (ScZng1) and animals (DrZNG1) and the uncharacterized *A. thaliana* AT1G26520 (AtZNG1) and *Chlamydomonas reinhardtii* Cre01.g052000 (CrZNG1) (**Figure 1**). The second subcluster that we have named ZNG2 (**Figure 1**) forms a monophyletic clade that is specific to plant and algal proteins (**Figure 2**). An additional small cluster, labeled cluster “4”, is composed entirely of proteins from Streptophyta, including both streptophyte algae (Charales and Klebsormidiales) and land plants (Mesangiospermae, Marchantiales, and Polypodiales). Unlike ZNG1 and ZNG2, cluster 4 proteins are not conserved throughout the land plant lineage but are related to orthologs found in chlorophyte and protist algae.

**Figure 3:**
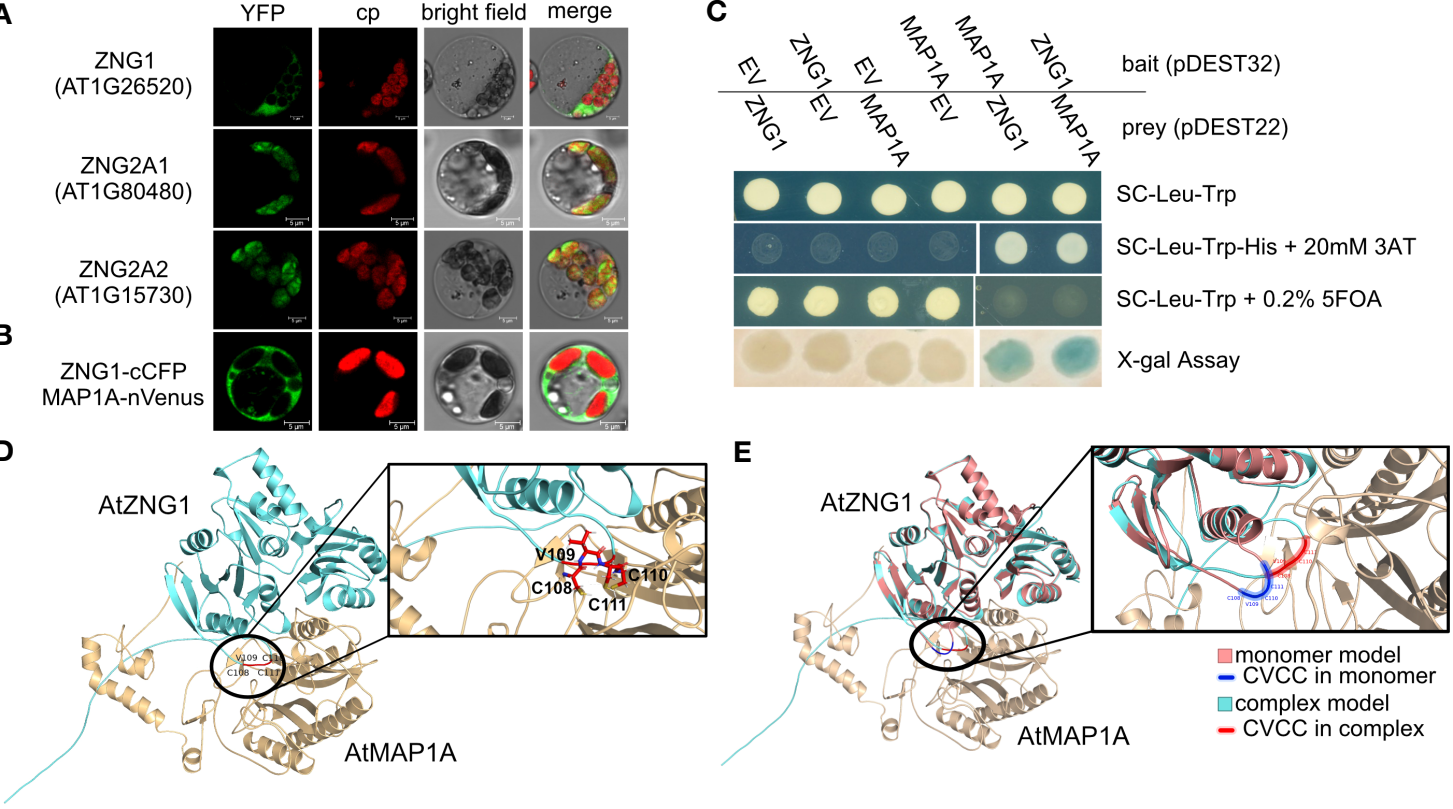
Interaction of AtZNG1 with AtMAP1A. **(A)** Confocal microscope images of AtZNG1, AtZNG2A1, and AtZNG2A2 fused to YFP on their C-terminus. Scale bar: 5 µm. **(B)** BiFC analysis. At*ZNG1* fused to the C-terminal fragment of blue fluorescent Cerulean protein complement (cCFP) and At*MAP1A* was fused with the N-terminal fragment of Venus (nVenus). Scale bar: 5 µm. **(C)** Yeast-two-hybrid assays. The transformants were grown on different selection media as indicated; X-gal assay was also performed. EV, Empty Vector. **(D)** Alphafold predicted structure of AtZNG1 (cyan)-AtMAP1A (wheat), with position of CxCC motif (red) in inset depicting all three Cys side chains oriented towards MAP1A (wheat). **(E)** Alphafold-predicted structure of AtZNG1 (salmon) with CxCC motif (blue), superimposed on the predicted structure of AtZNG1 (cyan) - AtMAP1 (wheat) complex with CxCC motif (red) to show relative conformation change between AtMAP1 bound (cyan) versus unbound AtZNG1 structure (salmon).

Based on phylogenetic reconstruction of proteins from the ZNG cluster, ZNG1 and ZNG2 form separate monophyletic clades (**Figure 2**). As with most every other eukaryotic genome, ZNG1 is almost always found in single-copy in algal and plant genomes. During the evolution of the ZNG2 subfamily, which is not found in the fungal or animal lineages, a duplication event early in Angiosperm evolution led to two paralogous groups: ZNG2A and ZNG2B (**Figures 2**, **S7**). Algal genomes encode a single ZNG2 protein, such as Cre16.g692901 from *C. reinhardtii*. The *A. thaliana* genome appears to have lost ZNG2B (**Figures 2**, **S7**). Instead, *A. thaliana* and other Brassicaceae have two ZNG2A paralogs: ZNG2A1 (AtZNG2A1; AT1G80480) and ZNG2A2 (AtZNG2A2; AT1G15730). There does not appear to be a correlation between loss of ZNG2B and duplication of ZNG2A. Also unlike ZNG1 that localizes to the cytosol, ZNG2 proteins from *A. thaliana* localize to the chloroplast (**Figure 3A**), perhaps explaining their uniqueness in algae and land plants, and suggesting a biological function in metal homeostasis within the plastid. Both *A. thaliana* ZNG2 proteins have long histidine stretches enriched in aspartic acid (AtZNG2A1) or glutamic acid (AtZNG2A2) between the GTPase and C-terminal domains (**Figure S2**).

In both the SSN and the phylogenetic tree, there is a closely related but separate clade of algal proteins in the ZNG cluster, represented by ZCP1 from *C. reinhardtii* (Hsieh et al., 2013; Malasarn et al., 2013) and ZCRP-A from the diatom *Phaeodactylum tricornutum* (Kellogg et al., 2022) (**Figures 1** and **2**). Like ZNG1, ZCP1 and ZCRP-A are linked to the Zn-deficiency response (Hsieh et al., 2013; Malasarn et al., 2013; Kellogg et al., 2022). There are some non-algal members of this subfamily, which may be the result of marine community horizontal gene transfer, including ZNG1-like genes in planktonic archaea from Candidatus Poseidoniales that are thought to have evolved from a photoheterotrophic ancestor (Rinke et al., 2019).

### 3.2 AtZNG1 interacts with AtMAP1A and loss leads to Zn-deficiency related phenotypes

Given the orthologous relationships across the eukaryotic ZNG1 family, we hypothesized that the molecular and biological functions of ZNG1 described in yeast and animals are conserved in plants. Consistent with this hypothesis, AtZNG1 is located in the cytosol (**Figure 3A**) and physically interacts with the cytosolic type I methionine aminopeptidase AtMAP1A based on both BiFC and Y2H assays (**Figures 3B, C**) but does not interact with the type II methionine aminopeptidases AtMAP2A and AtMAP2B (**Table S5**). The type I MetAPs are Zn dependent (Walker and Bradshaw, 1998), while the type II MetAPs are Mn dependent (Wang et al., 2003). Because of the difficulty in purifying a sufficient concentration of AtZNG1 for structural studies, we used AlphaFold to explore a possible complex between AtZNG1 and AtMAP1A, as was performed previously for ScZNG1 with ScMAP1 (Humphreys et al., 2021; Pasquini et al., 2022). The physical interaction between the *A. thaliana* proteins, identified with BiFC and Y2H assays (**Figures 3B, C**), is further supported by structural modeling of the AtZNG1 and AtMAP1A complex (**Figure 3D**), which is consistent with the previously published predicted ScZng1-ScMap1 complex (Humphreys et al., 2021). Further, superposition of the AtZNG1 model onto the AtZNG1-AtMAP1A complex suggests movement of the CxCC loop toward the active site of AtMAP1 when the complex is formed (**Figure 3E** and **Supplemental Movie 1**). A similar phenomenon was also observed for ScZng1 compared to the ScZng1-ScMap1 complex (**Supplemental Movie 2**). These models support formation of a functionally relevant AtZNG1-AtMAP1A complex and provide a mechanistic hypothesis for the transfer of Zn from AtZNG1 to AtMAP1A.

**Figure 4:**
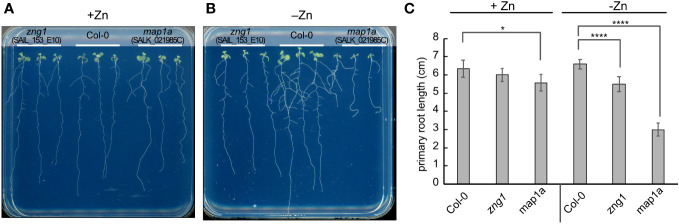
Impact of Zn deficiency on the *zng1* and *map1a* mutants. Seedlings (10 DAS) were grown on agarose-solidified medium with **(A)** or without **(B)** supplemented Zn, as indicated. Representative images are given. **(C)** Average primary root length for each genotype and growth condition. An average of 9 seedlings is given; three plates with three plants for each genotype per plate, grown at the same time in the same incubator. Error bars represent the standard deviation. **** indicates a P value < 0.0001, and * indicates a P value <0.05, as calculated with a Student’s T-test.

To better understand the function of AtZNG1 in planta, we grew Col-0, *zng1* (SAIL_153_E10), and *map1a* (SALK_021985C) in standard half-strength MS medium (referred to throughout as “plus Zn”) or in the same medium without added Zn (referred to throughout as “minus Zn”). Based on the expression of sentinel genes in the Zn-, Cu-, and Fe-responses and genes previously found to be highly induced upon Mn deficiency (Rodríguez-Celma et al., 2016), growth in the Zn-dropout medium results specifically in a Zn-deficiency response (**Figure S6**). Using growth as a proxy for N-terminal methionine excision (NME) activity, AtMAP1A does not appear to make a major contribution to NME in standard medium (**Figure 4A**), as reported previously (Ross et al., 2005). AtMAP1A is one of three cytosolic methionine aminopeptidases (MetAPs), and, based on previous studies, AtMAP2A and AtMAP2B contribute the majority of NME activity (Ross et al., 2005). However, we observed that in minus Zn, the *map1a* mutant grew poorly (**Figure 4B**), suggesting a greater dependency on AtMAP1A for cytosolic NME during Zn deficiency compared to Zn-replete conditions. Consistent with the hypothesized role of AtZNG1 in activating AtMAP1A, the *zng1* mutant was not significantly different from Col-0 in plus Zn, but in minus Zn, a growth defect was evident (**Figures 4A, B**). Compared to Col-0, the *zng1* mutant had slightly shorter primary and lateral roots (**Figure 4C**), whereas the *map1a* mutant was severely growth inhibited. Additionally, whereas the root architecture of Col-0 resembles foraging behavior that is observed for other nutrient deficiencies (Giehl and von Wirén, 2014), the *zng1* mutant does not have the same response (**Figure 4A**). Similar results were obtained from other mutant alleles of At*ZNG1* (SALK_203432C) and At*MAP1A* (SALK_097303C) (**Figure S8**). However, the T-DNA insert in SALK_203432C likely also disrupts the upstream gene At1g26530, which overlaps with the first two exons of *ZNG1* (**Figure S4**).

**Figure 5:**
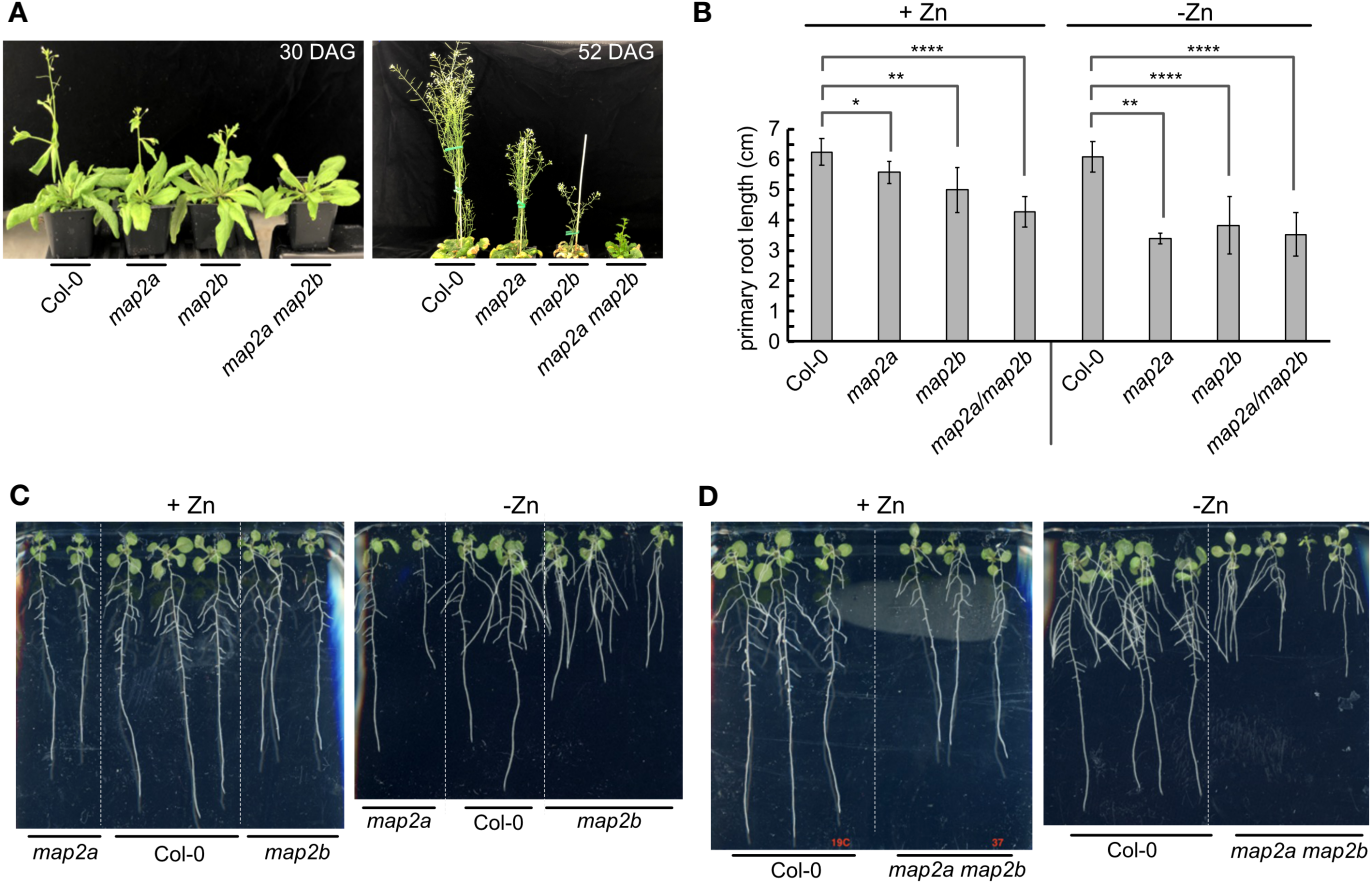
Impact of Zn deficiency on the *map2* mutants. **(A)** Soil-grown Col-0 and *map2* mutants at 30 days after germination (30 DAG) and 52 DAG. **(B)** Average length of primary roots for the indicated lines at 10 DAS in Phytagel-solidified medium with or without added Zn. Error bars represent the standard deviation of 9 seedlings (three plates with 3 plates on each plate, grown at the same time in the same incubator). **** indicates a P value < 0.0001, ** indicates a P value < 0.01, and * indicates a P value <0.05, as calculated with a Student’s T-test. **(C)** Representative images of plants measured for panel **(B)** Seedlings of Col-0 and single *map2* mutants (10 DAS) were grown on Phytagel-solidified medium with or without supplemented Zn, as indicated. **(D)** Representative images of plants measured for panel **(B)** Seedlings of Col-0 and double *map2* mutants (10 DAS) were grown on Phytagel-solidified medium with or without supplemented Zn, as indicated.

Since the *map1a* mutant exhibited sensitivity to Zn deficiency, we also tested whether the *map2a* and *map2b* mutants are sensitive. Loss of AtMAP2A and AtMAP2B activity is detrimental when plants are grown in soil (**Figure 5A**), showing an additive effect in the double mutant (**Figure 5A**), which was also observed on plates (**Figures 5C, D**). These results are expected given previous studies using fumagillin, a pharmacological inhibitor of MetAP type II (Ross et al., 2005). Like with the *map1a* mutant, the observed growth defects were exacerbated in minus Zn (**Figures 5B-D**). The lateral roots of the *map2b* and *map2a map2b* mutants were often as long or longer than the primary root (**Figures 5C**, **D**).

If AtZNG1 is needed for the activity of AtMAP1A during Zn deficiency, we reasoned that a *map2a map2b zng1* triple mutant would have a more severe fitness defect in minus Zn compared to either the *zng1* mutant or the *map2a map2b* mutant. This logic is based on the premise that in the *map2a map2b* mutant, cytosolic NME is dependent solely on AtMAP1A activity. Since loss of all MetAP activity would be embryo lethal (Ross et al., 2005), impairment of AtMAP1A activity would manifest as a growth defect. As such, growth of the *map2a map2b* mutant can be used as a proxy of AtMAP1A activity. When the *zng1* mutant was used as the mother in the cross with the *map2a map2b* mutant, only 1 seedling was found to be heterozygous at the *zng1* allele (*zng1*(+/-) *map2a map2b*), and this single plant did not produce seeds. When the *zng1* mutant was used as the father in the cross with the *map2a map2b* mutant, *map2a map2b zng1*(+/-) plants had severely delayed development and possible ovule abortion (**Figure S9**). As such, we were unable to attain a homozygous *map2a map2b zng1* triple mutant. Since loss of all MetAP activity would be embryo lethal (Ross et al., 2005), the inability to identify a *map2a map2b zng1* triple mutant suggests that loss of AtZNG1 results in synthetic lethality because AtMAP1A activity is compromised.

### 3.3 Loss of *ZNG1* impacts the transcriptome of roots

**Figure 6:**
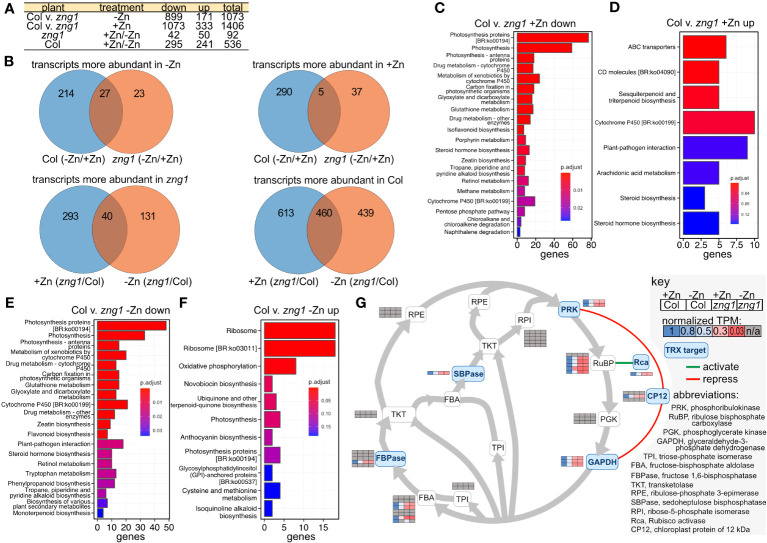
The *zng1* mutation leads to transcriptome alterations across multiple processes. **(A)** Description of sample comparisons involving Columbia (Col) and the *zng1* mutant with added Zn (+Zn) or minus Zn (-Zn). “Down” refers to transcripts with decreased transcript abundance and “up” refers to transcripts with higher transcript abundance. **(B)** Venn diagrams for visualizing the overlaps in the comparisons listed in **(A)**. **(C, D)** Top 20 significant KEGG pathways based on comparing the down-regulated and up-regulated genes in root samples between the Col and *zng1* mutant with added Zn (Col v. *zng1*). **(E, F)** Top 20 significant KEGG pathways based on comparing the down-regulated and up-regulated genes in root samples between the Columbia (Col) and *zng1* mutant without added Zn. **(G)** Pathway map of the Calvin-Benson-Bassham cycle. Squircles are used to indicate enzymes in the pathway (abbreviations can be found in the key). Heat maps of normalized TPM values are used to indicate transcript abundance for genes for each enzyme. Grey shading indicates that the transcript abundance for that gene did not differ between samples. Genes, fold changes, and TPM values can be found in **Table S7**. Thioredoxin (TRX) targets are shaded blue. A green line indicates that Rca activates RuBP, while red lines indicate that CP12 represses PRK and GAPDH. “n/a” is used to indicate the absence of a statistically significant difference between Col-0 and *zng1*. Data can be found in **Table S7**.

**Figure 7:**
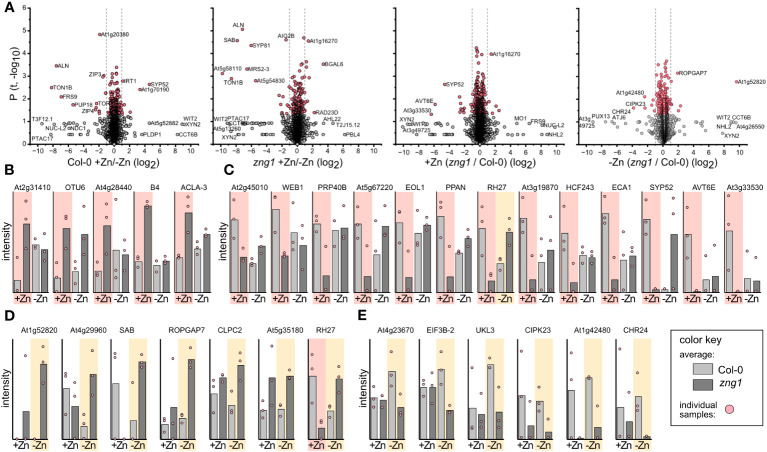
Impact of *zng1* mutation on the proteome. **(A)** Volcano plots of identified proteins in each comparison. Grey circles represent protein abundance changes with a P value > 0.05. Dotted lines represent the 2-fold cutoff. **(B)** In the presence of Zn, proteins that are higher in abundance in *zng1* (P value <0.5, FC>2). **(C)** In the presence of Zn, proteins that are higher in abundance in Col-0 (P value <0.5, FC>2). **(D)** In the absence of Zn, proteins that are higher in abundance in *zng1* (P value <0.5, FC>2). **(E)** In the absence of Zn, proteins that are higher in abundance in Col-0 (P value <0.5, FC>2). Note that RH27 is duplicated in panel **(C, D)**.

To better understand the impact of AtZNG1 and potential compensation mechanisms due to its loss, we employed RNA-seq (**Figure 6**; **Table S7**) and proteomics with Tandem Mass Tag (TMT) quantitation (**Figure 7**, **Table S6**) on root tissue from Col-0 and *zng1.* In Col-0, we identified 536 transcripts that were differentially abundant in roots from +Zn and -Zn growth conditions. Of these, 295 transcripts decreased in abundance in -Zn compared to +Zn, while 241 increased (**Figure 6A**), including the previously characterized transporters from the Zinc/Iron Permease (ZIP) family: ZIP1 (8.7 fold), ZIP3 (7.1 fold), ZIP4 (12.1 fold), ZIP5 (4.9 fold), ZIP9 (11.3 fold) and IRT3 (4.6 fold). The transcripts encoding these transporters were also increased in abundance in *zng1* in minus Zn compared to plus Zn, with no significance difference between *zng1* and Col-0 in minus Zn (**Table S7**). Comparing Col-0 with the *zng1* mutant, a large number of transcripts were differentially abundant (**Figure 6A**). In the presence of Zn, 333 transcripts were more abundant in *zng1*, while 1,073 were less abundant. In the absence of Zn, 899 transcripts were less abundant in *zng1*, while 171 were more abundant. Correlating with shorter roots in the *zng1* mutant, zeatin (a plant growth hormone produced in the roots) biosynthesis is decreased in both the presence and absence of Zn (**Figure 6**). Surprisingly, chloroplast-encoded transcripts and genes related to photosynthesis were enriched in the *zng1* mutant (**Figure 6**; **Table S7**).

After ranking DEGs by statistical significance (padj), in the top 30 most statistically significant more abundant *zng1* transcripts (compared to Col-0) in -Zn, half are chloroplast-genome-encoded ribosomal proteins. While proteins encoded by the top 30 less abundant transcripts include nuclear-encoded light-harvesting-related proteins (9 genes), proteins involved in carbon fixation (10 genes; including 4 rubisco subunits and a rubisco activase), and two RNA-binding proteins that are involved in regulating plastid-localized mRNAs. Intriguingly, many of the Calvin-Benson-Bassham cycle transcripts that are reduced in *zng1* compared to Col-0 encode those enzymes and regulatory proteins in the cycle that are regulated by thioredoxin (Michelet et al., 2013): FBPase, SBPase, PRK, GAPDH, Rubisco activase, and CP12 (**Figure 6G**). Genes involved in glutathione metabolism are also enriched in the list of reduced transcripts in the *zng1* mutant grown in either plus or minus Zn, suggesting that there may be dysregulation of redox control. Most of these transcripts related to photosynthesis are co-expressed with lower transcript abundance in the *zng1* mutant compared to Col-0 regardless of Zn, and enrichment of ribosomes, photosynthesis, and carbon fixation are captured in enrichment analysis (**Figure 6**). Although the RNA was extracted from roots and the overall abundances for these transcripts are significantly higher in the shoot samples, these results suggest pleiotropic impacts on the plastid and, potentially, defects in retrograde signaling, where expression from the plastid genome for ribosome synthesis is increasing but expression from the nuclear genome for light harvesting and carbon fixation is decreased in the *zng1* mutant.

### 3.4 Loss of *ZNG1* impacts the root proteome

Root proteomes from *zng1* and Col-0, grown under either standard conditions or Zn deprived, were analyzed by bottom-up mass spectrometry proteomics (Aebersold and Mann, 2003), and detected proteins were relatively quantified using tandem-mass-tags (Thompson et al., 2003). This approach identified a total of 3,837 proteins from the analyzed samples. Of these, 219 proteins were differentially abundant in Col-0 when comparing seedlings grown with added Zn (+Zn) or without added Zn (-Zn) (**Table S6**). Previously characterized Zn transporters (3 out of the 6 identified with RNA-seq), ZIP4 (5.5x fold change comparing -Zn/+Zn), ZIP3 (2.6x fold change comparing -Zn/+Zn), and IRT3 (1.3x fold change comparing -Zn/+Zn), were more abundant during Zn deficiency, while the Fe-uptake proteins IRT1 (2.1x fold change comparing +Zn/-Zn) and FRO2 (1.7x fold change comparing +Zn/-Zn) were less abundant, validating that the seedlings are experiencing Zn deficiency. We next asked which proteins are more or less abundant in *zng1* compared to WT in either +Zn or -Zn to identify any potential molecular processes impacted by the loss of AtZNG1 at the protein level.

In the presence of added Zn, we identified 181 proteins more abundant in Col-0 (**Table S6**). GO analysis of biological processes suggested enrichment in establishment of localization in cell (P value 5.68E-15), vesicle organization (P value 4.47E-02), and chromatin organization (P value 4.79E-02) among others (**Table S6**). In contrast, 144 proteins were found to be more abundant in *zng1*, including proteins involved in translation (P value 1.52E-06), such as multiple ribosomal protein subunits. Only 18 protein abundances changed more than 2-fold between the two lines (**Figures 7B, C**). Of these, three are potentially related to vesicle-mediated transport and cytokinesis, suggesting potential impacts on root growth in the *zng1* mutant in the presence of Zn: SYP52, a syntaxin from the SNARE family, ECA1, a clathrin assembly protein, and At3g19870, a putative AP-5 complex subunit beta-1 (adaptor protein for clathrin) and two proteins are related to chromatin: EOL1 is only expressed in rapidly growing roots cells and maintains H3K27 methylation, and OTU6/OTLD1 is an otubain-like histone deubiquitinase that represses gene expression through chromatin modification (**Figure 7**). The reciprocal abundance of these two proteins (EOL1 less abundant and OTU6 more abundant in the *zng1* mutant) may also reflect impacts on cell root growth.

In the absence of added Zn, we identified 109 proteins more abundant in Col-0 (enrichment in organic acid metabolic process (P value 7.13E-04) and carboxylic acid metabolic process (P value 1.08E.03)), and 123 proteins that were more abundant in *zng1* (enrichment also in organic acid metabolic process (P value 1.41E-04) (**Table S6**). Only 13 protein abundances changed more than 2-fold (**Figures 7D, E**). Intriguingly among these is CIPK23, which is considered a signaling hub that controls various nutrient homeostasis networks, including Zn (Ródenas and Vert, 2021). The reduced abundance of CIPK23 may partially explain the *zng1*-specific impacts on organic acid metabolism (de Bang et al., 2021).

### 3.5 Loss of *ZNG2A1* and *ZNG2A2* results in Zn-deficiency growth defects

The presence of the ZNG2 family in green algae and land plants suggests the possibility that AtZNG2A1 and AtZNG2A2 could have similar and disparate functional aspects compared to the ZNG1 family. GTPase motifs are conserved, as is the presence of a CxCC motif between the Switch I and Walker B motifs, suggesting conservation of GTPase activity and metal-binding (**Figure S2**). Functional divergence has occurred with respect to subcellular localization (**Figure 3A**); AtZNG2A1 and AtZNG2A2 localize to the chloroplast, suggesting that they do not interact with cytosolic AtMAP1A, as is the case for AtZNG1. However, there are three MAP1 proteins whose transit peptides have been found to target GFP to the chloroplast, MAP1C (AT1G13270; formerly MAP1B), MAP1B (AT3G25740; formerly MAP1C), and MAP1D (AT4G37040) (Giglione et al., 2000), presenting the possibility that ZNG2 may activate one or more plastid-localized MAP1 proteins. To test this hypothesis, we performed genetic analyses, protein-protein interaction assays, and structural modeling.

**Figure 8:**
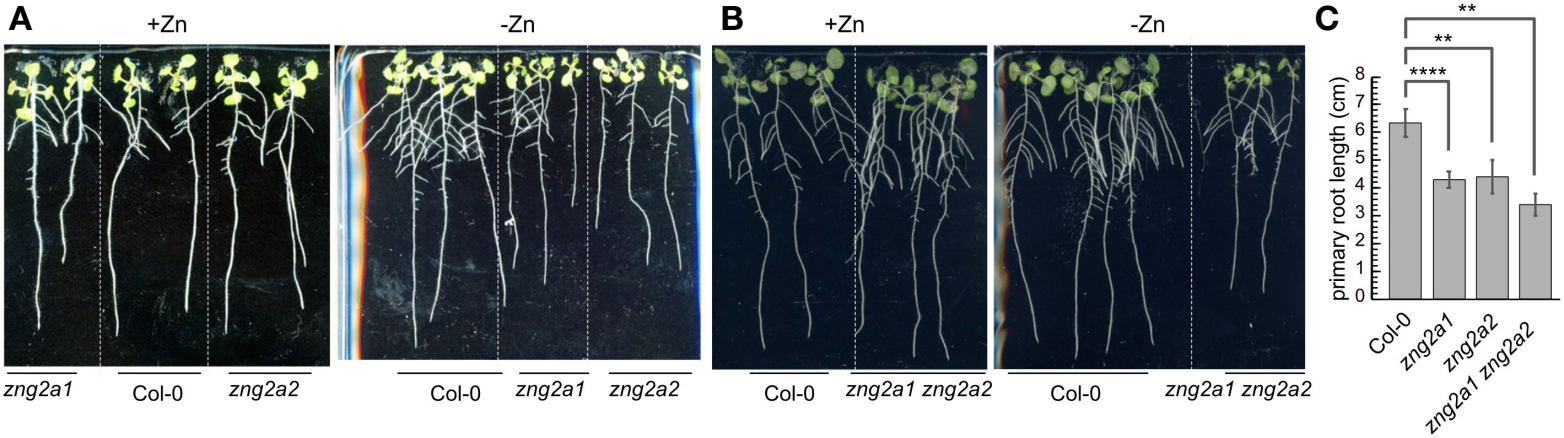
Impact of *zng2* mutation on growth with or without added Zn. **(A)** Comparison of single mutants with Col-0; 11 DAS **(B)** Comparison of Col-0 with the double mutant; 11 DAS. **(C)** Average primary root length in the absence of added Zn; 11 DAS. Error bars represent the standard deviation of 9 seedlings (three plates with 3 plates on each plate, grown at the same time in the same incubator). **** indicates a P value < 0.0001, ** indicates a P value < 0.01, as calculated with a Student’s T-test.

While no obvious growth defect was observed for *zng2a1* or *zng2a2* in the presence of Zn, both mutant lines were sensitive to Zn deficiency (**Figure 8A**). The effect of gene loss was additive, as disruption of both genes resulted in shorter primary roots during Zn deficiency compared to either single mutant (**Figures 8B, C**). These results point to a role for these plastid-localized proteins in enabling the plant to better acclimate to Zn deficiency. To test whether At*ZNG2A1* and At*ZNG2A2* may be involved in plastid NME, we attempted to generate various mutants with disruptions in plastidial NME. The *zng2a1 zng2a2 map1b* and *zng2a1 zng2a2 map1d* had no obvious growth defects compared to parental lines, but the *zng2a1 zng2a2 map1c* was not viable. These results could be due to an essential role of AtZNG2A1 and AtZNG2A2 in AtMAP1D and/or AtMAP1B activity, but we found that *map1b map1c* and *map1c map1d* mutants were not viable. Therefore, we could not test whether *zng2a1* or *zng2a2* may be involved in AtMAP1D or AtMAP1B activity, respectively.

Based on AlphaFold2 structures, AtZNG1, AtZNG2A1, and AtZNG2A2 are predicted to be similar at the tertiary level to one another and to the AlphaFold2 structure of ScZNG1 (**Figure S10**) but with several notable exceptions. Two small regions are found in ScZNG1 but absent from the *A. thaliana* proteins (**Figure S10**); one deletion (relative to ScZNG1) is between the Walker B and NKxD motifs of the GTPase domain, and the second deletion is in the C-terminal domain (**Figures S2**, **S10**). Compared to AtZNG1, AtZNG2A1 and AtZNG2A2 are predicted to have crucial secondary structure changes directly upstream of the CxCC residues, which are predicted to drastically impact the CxCC site (**Figure S10**). The positioning of the loop containing this CxCC site is hypothesized to be enable the metal transfer event to the target protein (as discussed in section 3.2). The secondary structure differences involve a transition of sheet-loop-sheet architecture for AtZNG1 to sheet-loop-helix for AtZNG2A1 and AtZNG2A2 (**Figure S10**). Also, in contrast to AtZNG1, the Alphafold2 predicted complex structures involving AtZNG2A1 or AtZNG2A2 with each of the plastid-localized MAP1 paralogs (AtMAP1B, AtMAP1C or AtMAP1D) failed to result in a biologically relevant complex and suggest non-specific interactions. Even at the predicted non-specific binding interface, the CxCC site of either AtZNG2A1 or AtZNG2A2 is not positioned in such a way to enable metal transfer to any of the MAP1 plastid-localized paralogs (**Figure S11**). Indeed, with Y2H assays, we were unable to detect a physical interaction between AtZNG2A1 or AtZNG2A2 with the plastid-localized MAP1 paralogs (**Table S5**).

### 3.6 Loss of *ZNG2A1* and *ZNG2A2* results in wide-ranging impacts on the transcriptome in shoots

**Figure 9:**
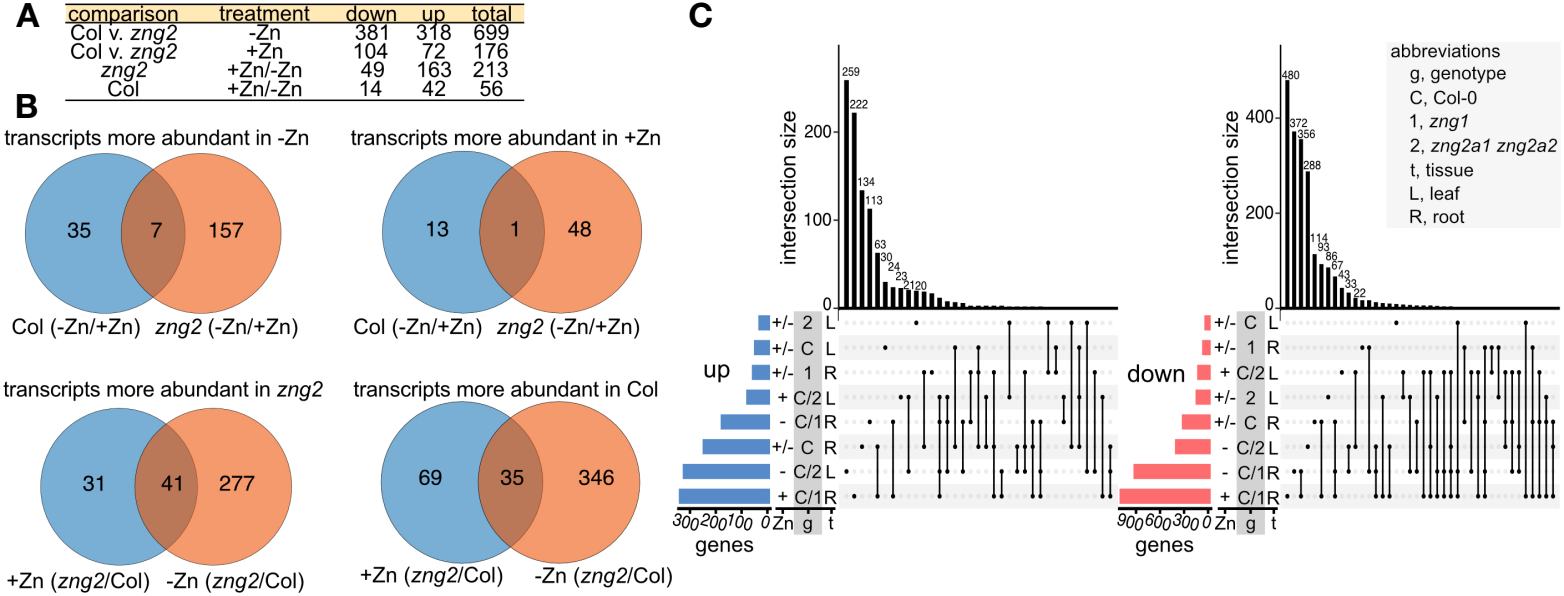
Overlaps among the RNAseq datasets. **(A)** Description of sample comparisons involving Columbia (Col) and the *zng2a1 zng2a2* mutant (“*zng2”* is used as an abbreviation) with added Zn (+Zn) or minus Zn (-Zn). “Down” refers to transcripts with decreased transcript abundance and “up” refers to transcripts with higher transcript abundance. **(B)** Venn diagrams for visualizing the overlaps in the comparisons listed in panel **(A, C)** UpSet plots showing the number of genes whose transcript abundance is increased (“up”) or decreased “down” in the analyzed comparisons from both RNAseq datasets. Intersection sizes smaller than 20 are not labeled. Abbreviations are given in the panel.

**Figure 10:**
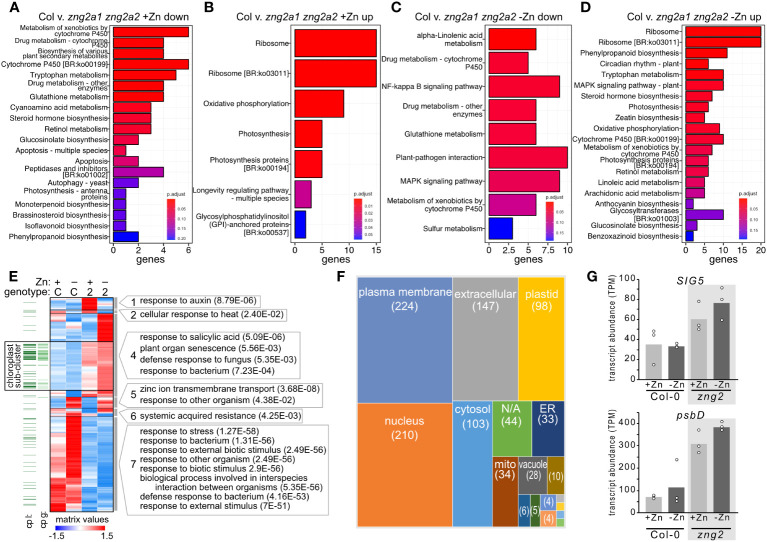
Impact of *zng2a1 zng2a2* mutation has a wide-ranging impact on the transcriptome with and without added Zn. **(A, B)** Top 20 significant KEGG pathways based on comparing the down-regulated and up-regulated genes in shoot samples between the Columbia (Col) and *zng2a1 zng2a2* mutant with added Zn (Col v. *zng2a1 zng2a2*). **(C, D)** Top 20 significant KEGG pathways based on comparing the down-regulated and up-regulated genes in shoot samples between the Columbia (Col) and *zng2a1 zng2a2* mutant without added Zn (Col v. *zng2a1 zng2a2).* **(E)** Heatmap of the 961 transcripts with statistically significant abundance differences in the comparisons between plus and minus Zn for the *zng2a1 zng2a2* mutant (labeled “2”), and the *zng2a1 zng2a2* mutant compared to Col-0 (labeled “C”) in either plus (labeled “+”) or minus Zn (labeled “-”). Proteins predicted to localize to the plastid “cp l” are indicated with a dark green line, while proteins encoded on the plastid genome “cp g” are indicated with a light green line. For each cluster, selected GO terms that are enriched (P value in parentheses) are shown. Cluster membership and order in the heatmap can be found in **Table S8**. **(F)** A box plot comparing the number of putative localizations for the proteins encoded by transcripts in panel **(E)**. “N/A” refers to proteins without a predicted localization in the Suba5 database. Boxes not labeled in the figure: brown, peroxisome; blue, golgi; green, cytosol/plasma membrane; light blue, vacuole/plasma membrane; orange, cytosol/nucleus; the unlabeled boxes have a single protein and represent extracellular/plasma membrane, mitochondrion/plastid, extracellular/endoplasmic reticulum, mitochondrion/cytosol, or golgi/plasma membrane. **(G)** Transcript abundance of *SIG5* and *psbD* in the analyzed RNA-seq samples. Corresponding data can be found in **Table S8**.

To better understand the *zng2a1 zng2a2* phenotype during Zn deficiency and since these proteins localize to the plastid, we employed RNA-seq on shoot samples and performed enrichment and co-expression analyses (**Figures 9** and **10**). In the presence of Zn, we identified 176 genes that had higher (72 genes) or lower (104 genes) transcript abundance in the *zng2a1 zng2a2* mutant compared to Col-0, while, in the absence of Zn, 699 genes had higher (318 genes) or lower (381 genes) transcript abundance in *zng2a1 zng2a2* mutant (**Figure 9**; **Table S8**). Based on enrichment analysis, in the presence of Zn, genes involved in secondary metabolism tended to be down-regulated, and ribosomal proteins and proteins involved in photosynthesis were up-regulated (**Figure 10**). The impact on ribosomes and photosynthesis was also observed in the absence of Zn (**Figure 10**). As many of these enrichment terms are shared with the *zng1* mutant root samples, they may reflect a general stress response, related to a shared physiological impact due to the absence of either the cytosolic or plastid-localized family members, and/or the result of an indirect impact of the *zng1* mutation on translation of AtZNG1A1 and AtZNG1A2. For instance, loss of *zng1* and consequential reduced AtMAP1A activity during Zn deficiency could impact the function of plastid-localized proteins that are translated on cytosolic ribosomes. Loss of the *zng2* proteins could indirectly impact the function of photosynthetic proteins synthesized in the plastid. However, other than the previously identified association of orthologous ZNG2 proteins with the transcription/translation machinery in the plastid, the direct mechanism of *zng2* loss on expression differences is unknown. Further, we did not observe many differentially abundant transcripts that were shared between the two RNA-seq experiments (**Figure 9C**), but since root tissue was collected from *zng1* and shoot tissue for *zng2a1 zng2a2*, the small overlap is not surprising.

Of the transcripts with statistically significant abundance differences (961 genes) in the comparisons between plus and minus Zn for the *zng2a1 zng2a2* mutant, and the *zng2a1 zng2a2* mutant compared to Col-0 in either plus or minus Zn (**Figure 9**), we identified 7 co-expression clusters (**Figure 10E**). The dominant expression patterns are genes with transcript abundances either reduced or increased in the mutant in both plus and minus Zn (**Figure 10E**). Out of the 799 genes with a statistically significant difference in transcript abundance in the *zng2a1 zng2a2* mutant compared to Col-0, only 88 encoded proteins are predicted to localize to the chloroplast (the presumed site of a direct defect due to the loss of AtZNG2) (**Figures 10E, F**). Half of these are chloroplast genome-encoded proteins. Of the nuclear genome-encoded plastid-localized proteins that are more or less abundant in the *zng2a1 zng2a2* mutant, there is an enrichment in starch catabolic process (P value 1.28E-2), jasmonic acid biosynthetic process (P value 5.92E-4), sulfate assimilation (P value 2.56E-02), and cellular response to light stimulus (P value 3.70E-02). On this list is also AtSIG5 (AT5G24120), a sigma factor that functions in the regulation of plastid genes. The increased expression of this sigma factor in the *zng2a1 zng2a2* mutant could at least partially explain the observed increased expression of plastid genes, such as *psbD* (**Figure 10G**). These and other transcripts encoding chloroplast-localized proteins are enriched in a sub-cluster of cluster 4 (**Figure 10E**), where transcript abundances are typically higher in the mutant compared to Col-0 (**Table S8**).

We also observed that Fe homeostasis may be disrupted in the *zng2a1 zng2a2* mutant or down regulated to compensate for physiological defects. In the presence of Zn, the 3 most statistically down-regulated transcripts encode AtCYP82C4 (a cytochrome P450 enzyme involved in synthesis of sideretin, involved in solubilizing Fe), AtIRT1 (Fe transporter), and AtNAS2 (nicotianamine synthase). Transcript abundance for AtCYP82C4 and AtIRT1 are also reduced in the absence of supplemented Zn (cluster 7), but AtNAS2 transcript abundance increases (cluster 5). AtNAS2 transcript abundance was previously found to increase in low Zn (Sinclair et al., 2018). Therefore, presumably the response to Zn deficiency is not perturbed in the *zng2a1 zng2a2* mutant, but the mutant appears to be responding at the transcript level as if iron uptake should be reduced.

## 4 Discussion

In the cytoplasm of eukaryotes, N-terminal methionine excision (NME) is performed by two different classes of MetAP with different cofactor requirements. MetAP1/MAP1 is a Zn-dependent enzyme (Walker and Bradshaw, 1998) whereas MetAP2/MAP2 in Mn dependent (Wang et al., 2003). In addition to cofactor dependence, these two classes of enzyme are also expected to differ in substrate specificity (Chen et al., 2002). Whether due to cofactor dependence, substrate specificity, or some other characteristic, the physiological role of these classes differs between organisms. In the yeast *S. cerevisiae*, MAP1 is the dominant class and loss of Sc*MAP1* leads to a severe growth defect (Chang et al., 1992), whereas in *A. thaliana* disruption of At*MAP1A* does not result in an obvious growth phenotype, and AtMAP2A/AtMAP2B dominate (Ross et al., 2005). However, NME appears to be universally essential, and loss of cytosolic NME (i.e., loss of type 1 and type 2 MetAPs) is lethal in both *A. thaliana* and *S. cerevisiae* (Li and Chang, 1995; Ross et al., 2005).

Based on our analysis of the MetAP mutants, the distinct physiological roles of these enzymes and the requirement for NME under different growth conditions appears to be more complex. For example, while loss of At*MAP1A* does not have an apparent growth impact under standard growth conditions, we observed that the mutant grows poorly in the absence of supplemented Zn. AtMAP2A and AtMAP2B are presumably present and functioning under this condition as well, since corresponding mutants also grow poorly in the absence of Zn. The results suggest that during Zn deficiency, both classes of MetAP are needed. The underlying reason for this requirement is not known and is likely a combination of molecular defects due to the role of MetAPs in translation fidelity (Fujii et al., 2018), free methionine or glutathione homeostasis (Frottin et al., 2009), and/or one more proteins that require i-Met cleavage for activity or to regulate protein stability (Kim et al., 2019). Nevertheless, AtMAP1A has a function during growth in low Zn, which cannot be performed by AtMAP2A/AtMAP2B. An interesting, but untested hypothesis, is that AtMAP1A has one or more substrates that are specifically needed during Zn deficiency, explaining the conditional phenotype. Further work is needed to pinpoint the physiological role and key substrates of AtMAP1A during Zn deficiency. Regardless of the reason for why loss of AtMAP1A leads to the observed phenotypes, the requirement for a Zn-dependent enzyme during Zn deficiency would necessitate mechanisms to ensure Zn binding. Thus, explaining the conservation of AtZNG1 in *A. thaliana* where MAP2, not MAP1, is the dominant type of methionine aminopeptidase under standard growth conditions (Ross et al., 2005).

The molecular and biological function of ZNG1 proteins appear to be largely conserved across the major lineages of eukaryotes. This conclusion is supported by the vertical inheritance of both ZNG1 and MAP1 since the last universal ancestor of eukaryotes, the conservation of a physical interaction in yeast, animals, and, as shown here, plants, and the conserved fitness impact during Zn deficiency caused by loss of ZNG1, which is tied to MAP1 function. These results suggest that the functional relationship between ZNG1 and cytosolic MAP1 evolved coincidently with the evolution of eukaryotes and has been maintained during eukaryotic diversification (with some exceptions, such as the nematode *Caenorhabditis elegans* that has since lost ZNG1). In the context of the broader nucleotide-dependent metallochaperone (NMC) family, where different NMCs have different target metalloproteins (Nojiri et al., 1999; Chandrangsu et al., 2019; Young et al., 2021), the specialization of ZNG1 as a Zn chaperone for MAP1 was a key event in the evolution of Eukaryota.

At the molecular level, the complex between ZNG1 and MAP1 appears to not have changed significantly over the billions of years of evolution since that time. In yeast, animals, and plants, an unstructured, flexible N-terminal extension in ZNG1 can interact with the N-terminal Zn-finger region of MAP1. Additionally, based on structural modeling, the CxCC loop of ZNG1 is positioned at the entrance to the metal-binding site of MAP1 and, further, as shown here, is predicted to move upon binding to MAP1. This conformational change that can occur in the presence of MAP1 is expected to alter Zn coordination and could be responsible for Zn transfer to MAP1. The connection between loop movement and GTP hydrolysis, which was previously found to be required for Zn transfer (Pasquini et al., 2022), is unknown, but the structural modeling suggests that binding to MAP1 is important for the transfer. No ZNG1 ortholog has yet-to-be structurally characterized experimentally, and the protein complex and structural details of the Zn-transfer event have yet to be experimentally verified. Regardless, the modeling data presented here provides several reasonable hypotheses for the molecular details of the conserved Zn-transfer reaction.

At the biological level, the impact of a MAP1 dysfunction, due to the loss of ZNG1, is expected to be different across eukaryotes. In *A. thaliana*, we observed that loss of At*ZNG1* impacts root growth, the size of leaves, and leads to pleotropic impacts on gene expression during Zn deficiency, which were measurable at the transcriptome and proteome levels. Many of these gene expression changes are expected to be a consequence of reduced AtMAP1A activity, including protein defects due to improper NME and likely translational defects, as observed in yeast (Fujii et al., 2018). Since the majority of plastid-localized proteins are translated on cytosolic ribosomes, where AtMAP1A binds and cleaves the iMet of target peptides, impacts on chloroplast and photosynthesis in the *zng1* mutant are not surprising. We also noticed some intriguing impacts on Calvin-Benson-Bassham cycle transcripts and glutathione metabolism that could be indicative of redox imbalance in the *zng1* mutant. Perturbation of glutathione homeostasis was previously linked to NME inhibition in *A. thaliana* (Frottin et al., 2009). At this point, however, it is difficult to differentiate between transcriptome and proteome impacts that are a direct result of improper NME and which are downstream compensation mechanisms. Indeed, proteins with reduced abundance in *zng1* are in enriched processes, such as localization in cell and chromatin organization, which likely would have far-ranging impacts on the rest of the proteome. These conclusions are also predicated on the assumption that the *zng1* phenotype during Zn deficiency is entirely due to dysfunction of AtMAP1A and not other as-of-yet unknown target proteins.

Whereas the evolution of ZNG1 coincided with the evolution of eukaryotes, ZNG2 evolution coincided with evolution of Archaeplastida. The two *A. thaliana* proteins, AtZNG2A1 and AtZNG2A2, localize to the chloroplast, and their absences result in sensitivity to Zn deficiency, presumably due to one or more plastidial defects in Zn homeostasis. The biological function of these proteins remains unknown. Conservation of functional motifs and a role in growth during Zn deficiency suggest that these proteins are also GTP-dependent metal chaperones involved in Zn homeostasis, like ZNG1. However, the Zn-dependent protein(s) they interact with in the plastid are unknown. We were unable to find evidence that one or both ZNG2 proteins in *A. thaliana* interact with a chloroplast-localized MAP1 protein. Indeed, the organelle-localized MAP1 (AtMAP1B, AtMAP1C, and AtMAP1D) and ZNG2 proteins lack the N-terminal protein extensions characteristic of the cytosol-localized homologs. These results support the possibility of a yet-to-be identified protein(s) in the plastid that requires AtZNG2A1 and/or AtZNG2A2 when plants experience Zn deficiency. As the majority of plastid-localized metalloproteins are imported as unfolded polypeptides (Jarvis, 2008), mechanisms for transporting and transferring metals to these metal-dependents proteins are required to exist in the plastid.

Previous co-purification of ZNG2 proteins with chloroplast expression machinery (Pfalz et al., 2006; Majeran et al., 2012) points to a functional connection to a Zn-dependent protein involved in transcription and/or translation in plastids. ZNG2 homologs are often called pTAC17, because a homolog co-purified with transcriptionally active chromosomes from mustard (*Sinapis alba*) chloroplasts (Pfalz et al., 2006). Supporting a functional connection to transcription and/or translation in plastids, a ZNG2 homolog was also identified in the nucleoid fraction of proplastids and chloroplasts isolated from maize (Majeran et al., 2012) and co-purifies with maize HCF173, a *psbA* translational activator that is known to bind *psbA* mRNA in the plastid (Watkins et al., 2020), while AtZNG2A1 (AT1G80480) co-purifies with LEFKOTHEA, a RNA-binding protein that participates in chloroplast group II intron and nuclear pre-mRNA splicing (Daras et al., 2019). A ZNG2 homolog from tobacco co-purified with the plastid-encoded RNA polymerase (PEP) complex (Suzuki et al., 2004), but since a Ni-charged affinity resin was used to purify a variant of tobacco PEP α subunit (RpoA) fused to a polyhistidine tag (His-tag), ZNG2 was likely co-purified as a contaminant that bound to the Ni of the resin through its His-rich loop, rather than as a protein that physically associates with RpoA. Based on transcriptomics analysis, as with *zng1*, we observed potentially pleiotropic consequences across the cell in the *zng2a1 zng2a2* mutant. Given the connections of ZNG2 homologs to transcription and/or translation, an untested hypothesis is that one or both ZNG2 proteins interact with another component of the plastid transcriptionally active chromosome, and the *zng2a1 zng2a2* defect during Zn deficiency is due to impaired transcription or translation. However, given the large number of transcripts that are differentially abundant in the mutant compared to the parent, it is difficult to pinpoint which metalloprotein(s) (presumably a Zn-dependent protein) in the chloroplast is defective in the mutant. Given the conservation of the ZNG2 family across Chlorophyta and Streptophyta and in some red algal genomes, presumably the target(s) are also conserved plastid-localized proteins.

## Data availability statement

The datasets presented in this study can be found in online repositories. The names of the repository/repositories and accession number(s) can be found in the article/**Supplementary Material**.

## Author contributions

CB-H, LZ, DW, and MX designed experiments. LZ performed the Y2H, phenotyping experiments, and tissue collection for RNA-seq and proteomics. JB performed RNA-seq data analysis. PC performed and analyzed proteomics experiments. AF performed RNA purifications for RNA-seq. AF and FH performed genotyping and crosses. MR performed RT-PCR of T-DNA insertion lines. KC performed protein structure predictions and analysis. MX and DT performed protoplast experiments. CB-H wrote the manuscript with input from all authors. All authors contributed to the article and approved the submitted version.

## Supplementary material

SUPPLEMENTARY TABLE 1 Description of nodes in sequence similarity network.

Supplementary Table 2 Data and information associated with the ZNG phylogenetic tree.

Supplementary Table 3 Data associated with the plant ZNG1 and ZNG2 phylogenetic trees.

Supplementary Table 4 Primer sequences used to confirm mutant alleles.

Supplementary Table 5 Description of yeast-two-hybrid (Y2H) constructs and data.

Supplementary Table 6 Relative protein abundance, fold changes, and statistics for Col-0 and *zng1* roots from seedlings grown with and without added Zn.

Supplementary Table 7 TPM values, fold changes, and statistics for Col-0 and *zng1* roots from seedlings grown with and without added Zn.

Supplementary Table 8 TPM values, fold changes, and statistics for Col-0 and *zng2a1 zng2a2* shoots from seedlings grown with and without added Zn.

Supplementary Movie 1 The morph movie of AtZNG1 Alphafold-predicted structure superimposed on AtZNG1-AtMAP1A predicted complex, captures the transition of CxCC motif (red) of AtZNG1 (cyan) into the binding pocket of AtMAP1A (wheat). The movie was generated using ChimeraX.

Supplementary Movie 2 The morph movie of ScZNG1 Alphafold model (AF-P53729) superimposed on ScZNG1-ScMap1 predicted complex (DOI: 10.5452/ma-bak-cepc-0793) captures the transition of CxCC motif (red) of ScZNG1 (green) into the binding pocket of ScMap1(pink). The movie was generated using ChimeraX.
