# Supplementary material for: Two related families of metal transferases, ZNG1 and ZNG2, are involved in acclimation to poor Zn nutrition in Arabidopsis
Source: Front Plant Sci. 2023 Oct 25;14:1237722. doi: 10.3389/fpls.2023.1237722 (PMC10642216; doi:10.3389/fpls.2023.1237722)
Supplement: Supplementary file 1 [file DataSheet_1.pdf]

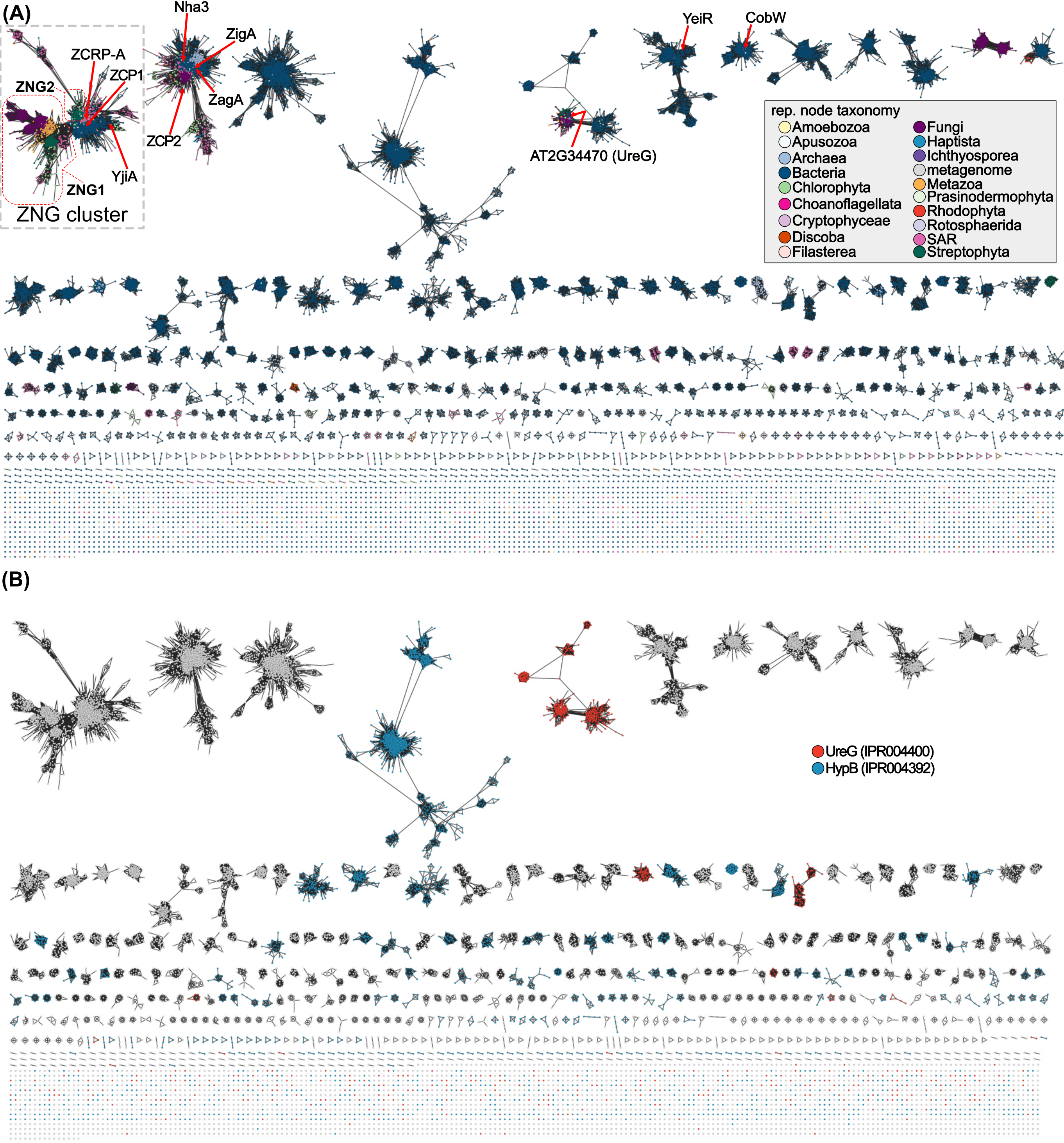

Figure S1. Sequence similarity network of proteins containing the CobW/HypB/UreG, nucleotide-binding domain (PF02492). **(A)** Nodes colored by taxonomy according to the color key. **(B)** Nodes colored by the presence of IPR004400 or IPR004392 according to the color key.

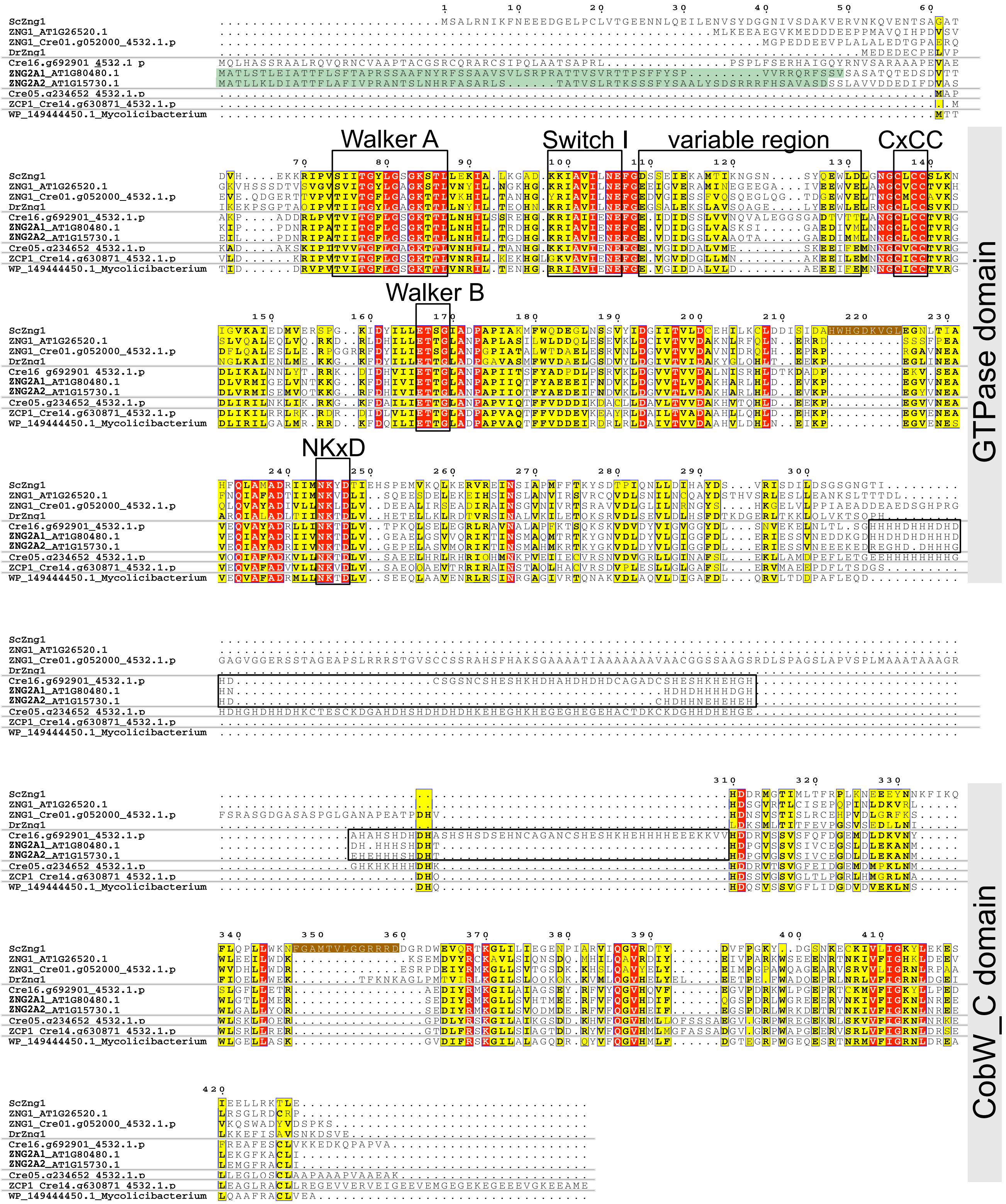

Figure S2. Multiple sequence alignment of ZNG1 and ZNG2 proteins. The full-length amino acid sequences of ZNG1 proteins from *Saccharomyces cerevisiae* (Sc), *Danio rerio* (Dr), *Arabidopsis thaliana*, and *Chlamydomonas reinhardtii* were aligned with ZNG2 proteins from *A. thaliana* and *C. reinhardtii*, two additional *C. reinhardtii* homologs (please note that green algae, including *C. reinhardtii*, have a relatively large number of ZNG-like proteins (not shown) that are from multiple evolutionarily distinct clades), and a similar ZNG-like protein from the bacterium *Mycolicibacterium* sp. P9-22. Horizontal grey lines are used to delineate orthologous groups. Vertical grey boxes delineate the GTPase domain from the CobW-C domain. Red column shading indicates 100% conserved amino acids and bold letters with yellow column shading indicates similar residues. Black boxes are used to highlight the histidine-rich stretches in the ZNG2 proteins, conserved GTPase motifs, and the variable region immediately upstream of the CxCC motif, which results in secondary structure differences between ZNG1 and ZNG2. Insertions unique to ScZng1 are highlighted with brown shading, and the predicted chloroplast-targeting transit peptides for AtZNG2A1 and AtZNG2A2 are highlighted with green shading.

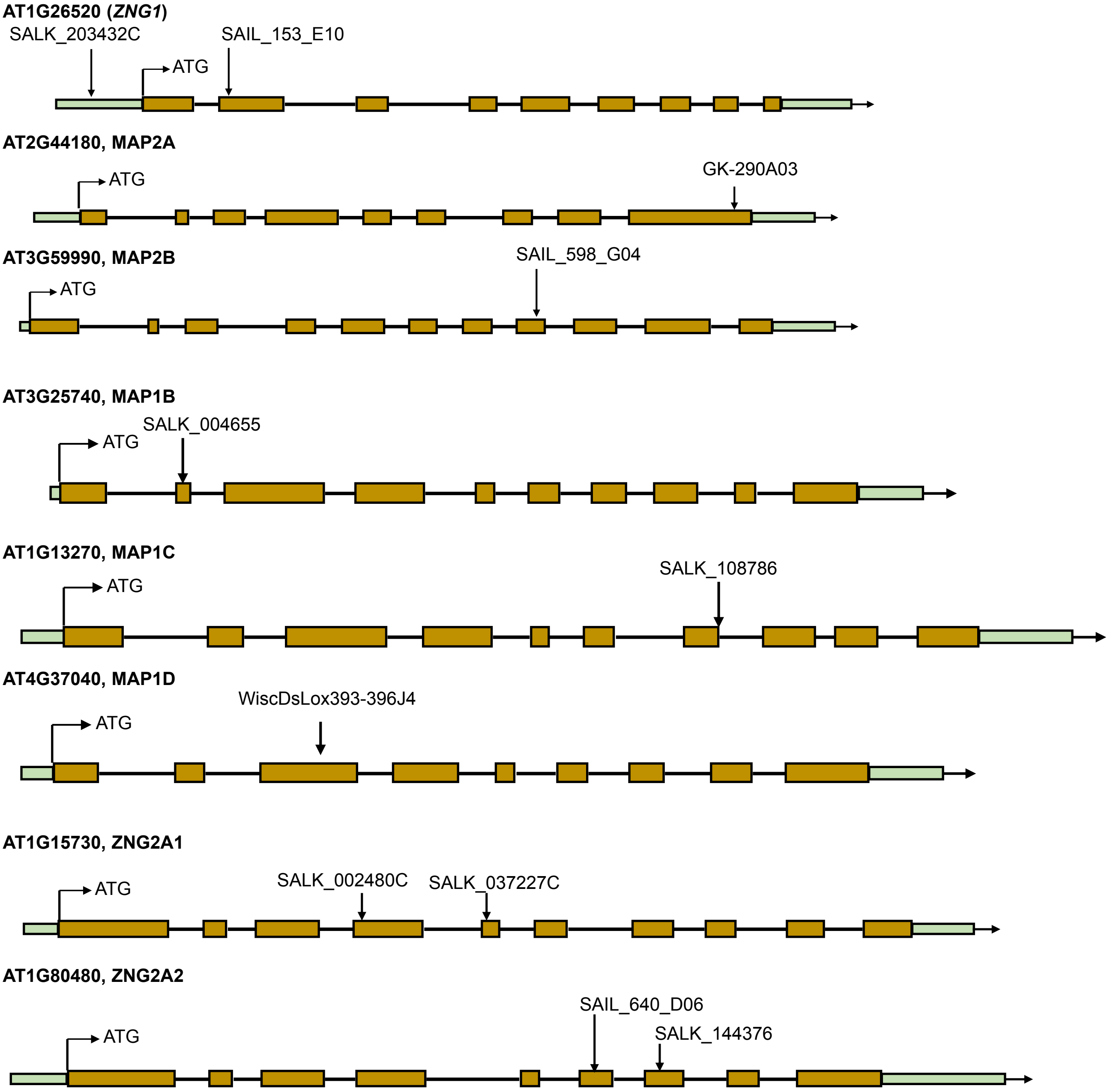

Figure S3. Sequencing-verified location of T-DNA inserts for mutants used in this study. For SAIL_598_G04, SALK_002480C, and SALK_037227C, the location of the T-DNA insert is misannotated in TAIR. The correct locations are shown here. Details for the *map1a* mutants can be found in Ross et al., (2005).

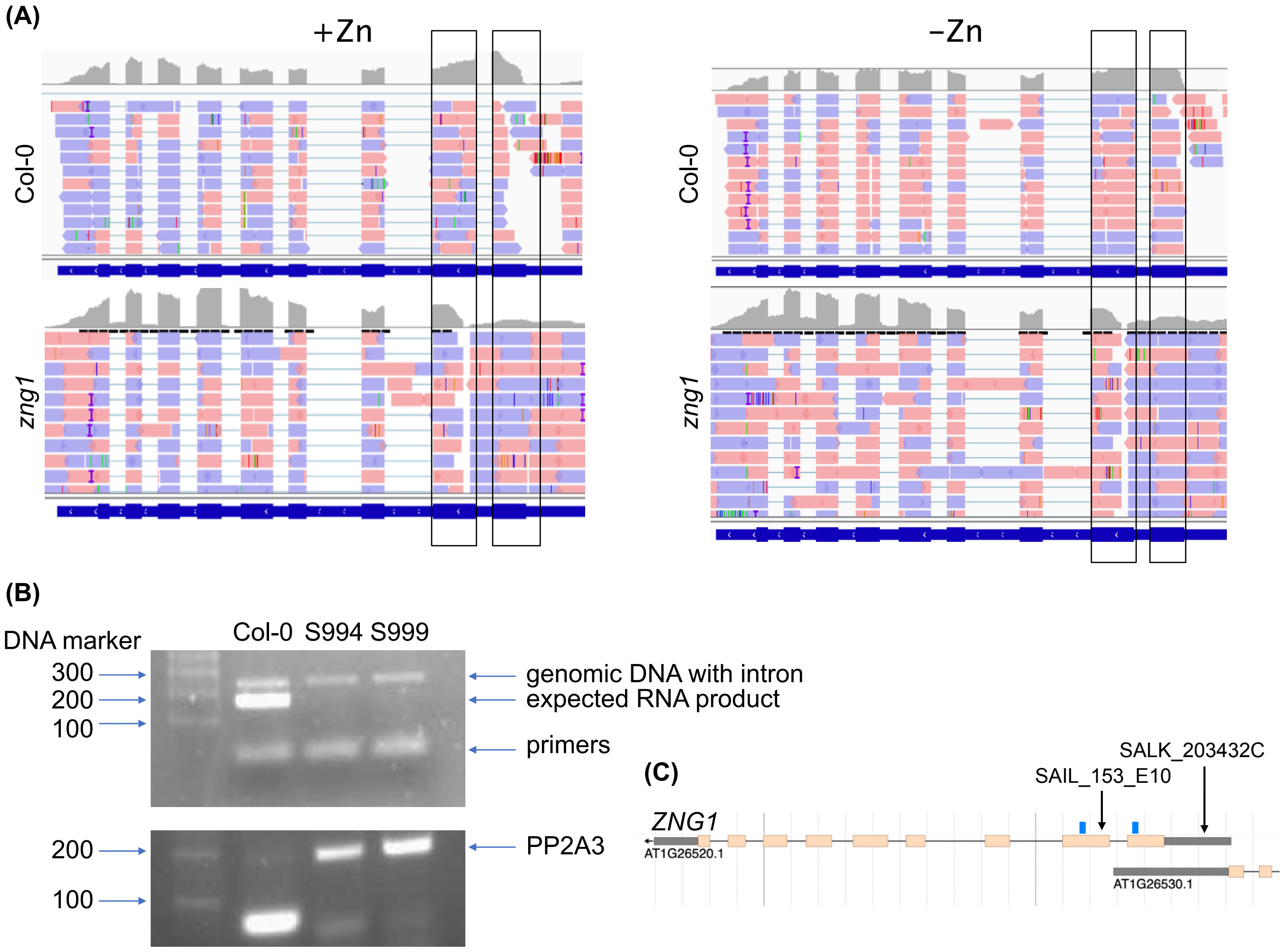

Figure S4. Analysis of *ZNG1* mRNA. **(A)** Browser view of RNA-seq reads aligned to *ZNG1* from either Columbia (Col-0, top) or the *zng1* mutant (bottom) from plants grown in either plus Zn (+Zn) or minus Zn (-Zn). In both growth conditions, there are far fewer reads aligned to the first exon and at the junction between the first and second exon; 5' of the T-DNA insert (Figure S3). Black boxes are used to compare the aligned reads (grey pileup) for Col-0 vs. *zng1*. The reads that are present at the 5' end of *ZNG1* in the *zng1* mutant are likely from the upstream gene At1G26530 that has higher transcript abundance in the *zng1* mutant compared to Col-0 (>10-fold more in *zng1* in both growth conditions). As shown in panel C, At1G26530 overlaps with the first exon of *ZNG1*. **(B)** Image of an agarose gel of PCR products from a cDNA preparation from Col-0 and the two *zng1* mutants analyzed in this study, SALK_203432C/S994 or SAIL_153_E10/S999. The RNA sample was not treated with DNAseI in order to amplify both genomic DNA and cDNA. *ZNG1* primers (CAGTTGGTGTGTCTGTTATCACTGGC and TCAACCCATTCTTCAACAATGGCACC). *PP2A3* (primers TGATGCAATCTCTCATTCCGATAG and AGCATGGCCGTATCATGTTCT) was used to confirm that the absence of amplification product corresponding to the expected cDNA product in the *zng1* mutants is not due to poor cDNA generation. **(C)** Screenshot of the Phytozome browser showing the *ZNG1* gene model. The locations where primers anneal are shown with blue rectangles, and arrows are used to show location of T-DNA inserts in the two mutants used in this study.

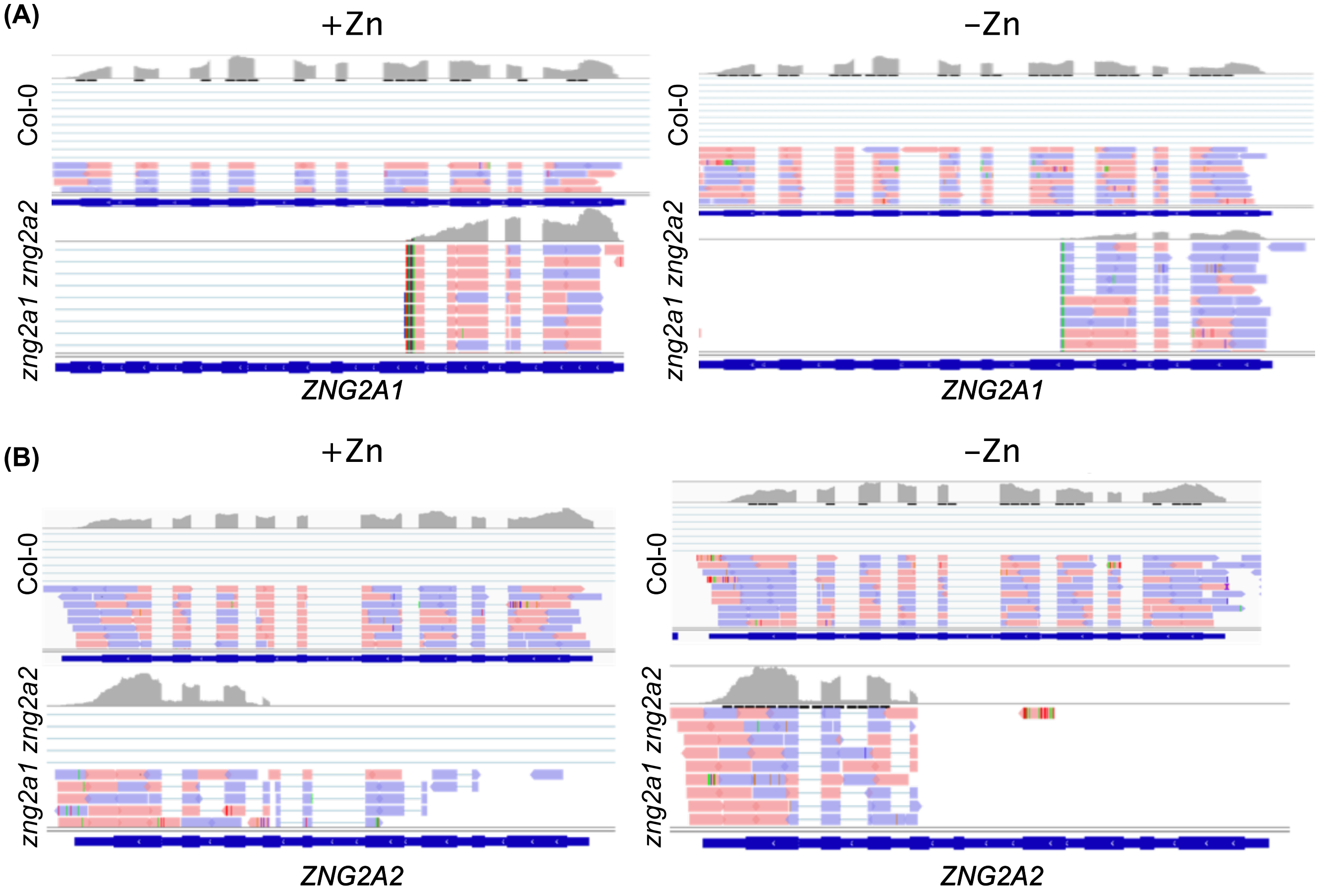

Figure S5. Browser view of RNA-seq reads aligned to *ZNG2A1* and *ZNG2A2*. **(A)** Alignment of RNA-seq reads to the *ZNG2A1* gene from either Columbia (Col-0, top) or the *zng2a1 zng2a2* mutant (bottom) from plants grown in either plus Zn (+Zn) or minus Zn (-Zn). In both growth conditions, there are far fewer reads after the third exon that is the location of the T-DNA insert (Figure S3). **(B)** Alignment of RNA-seq reads to the *ZNG2A2* gene from either Columbia (Col-0, top) or the *zng2a1 zng2a2* mutant (bottom) from plants grown in either plus Zn (+Zn) or minus Zn (-Zn). In both growth conditions, there are far fewer reads after the fourth exon.

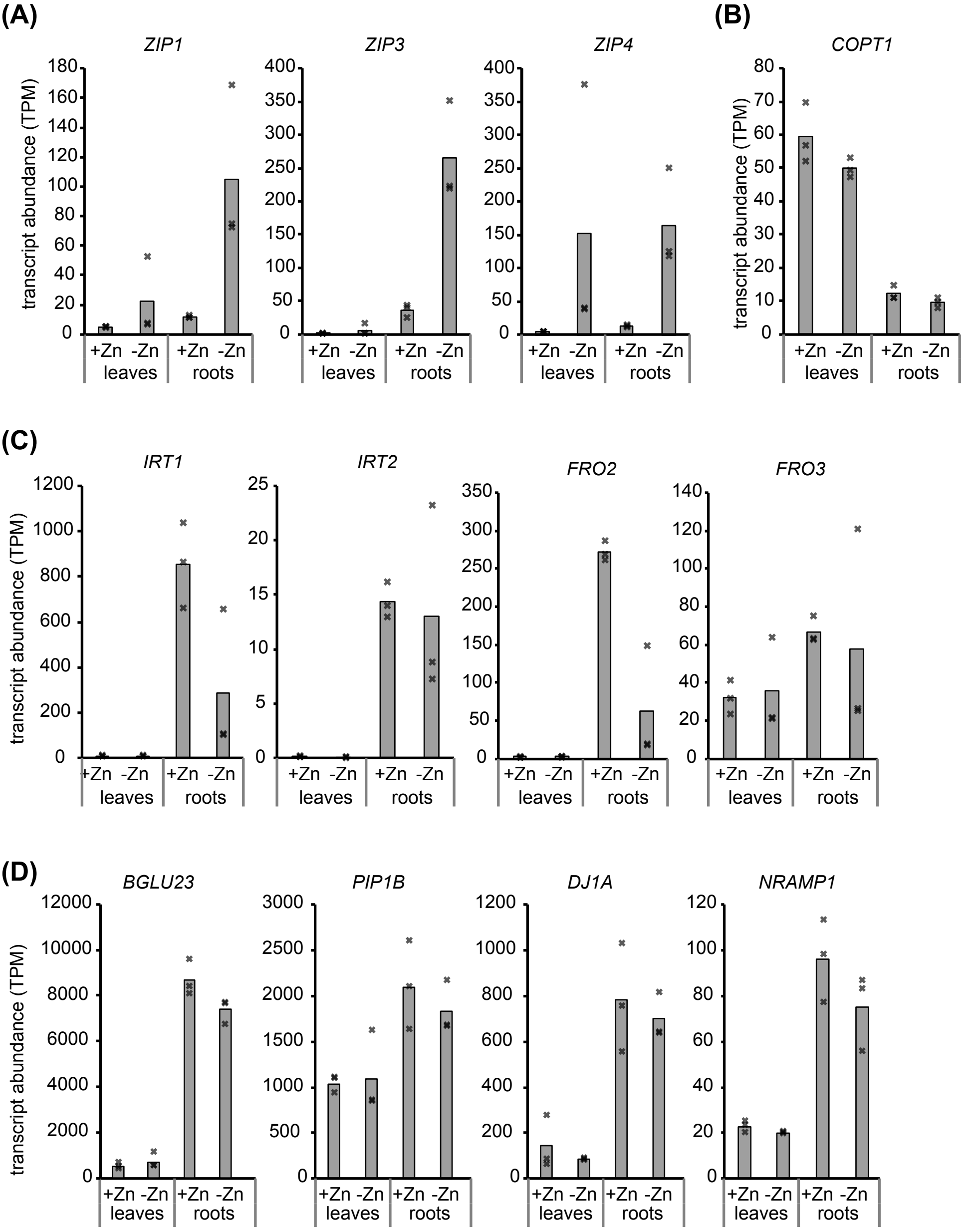

Figure S6. Transcript abundance of sentinel genes for the Zn-deficiency **(A)**, Cu-deficiency **(B)**, and Fe-deficiency **(C)** responses. **(D)** Transcript abundance for three genes previously found to be the most highly induced during Mn deficiency and NRAMP1 a Mn transporter. Bar represent averages from three biological replicates, and hatch marks represent individual replicates. "+Zn" refers to medium with supplemental Zn, and "-Zn" refers to medium without supplemental Zn.

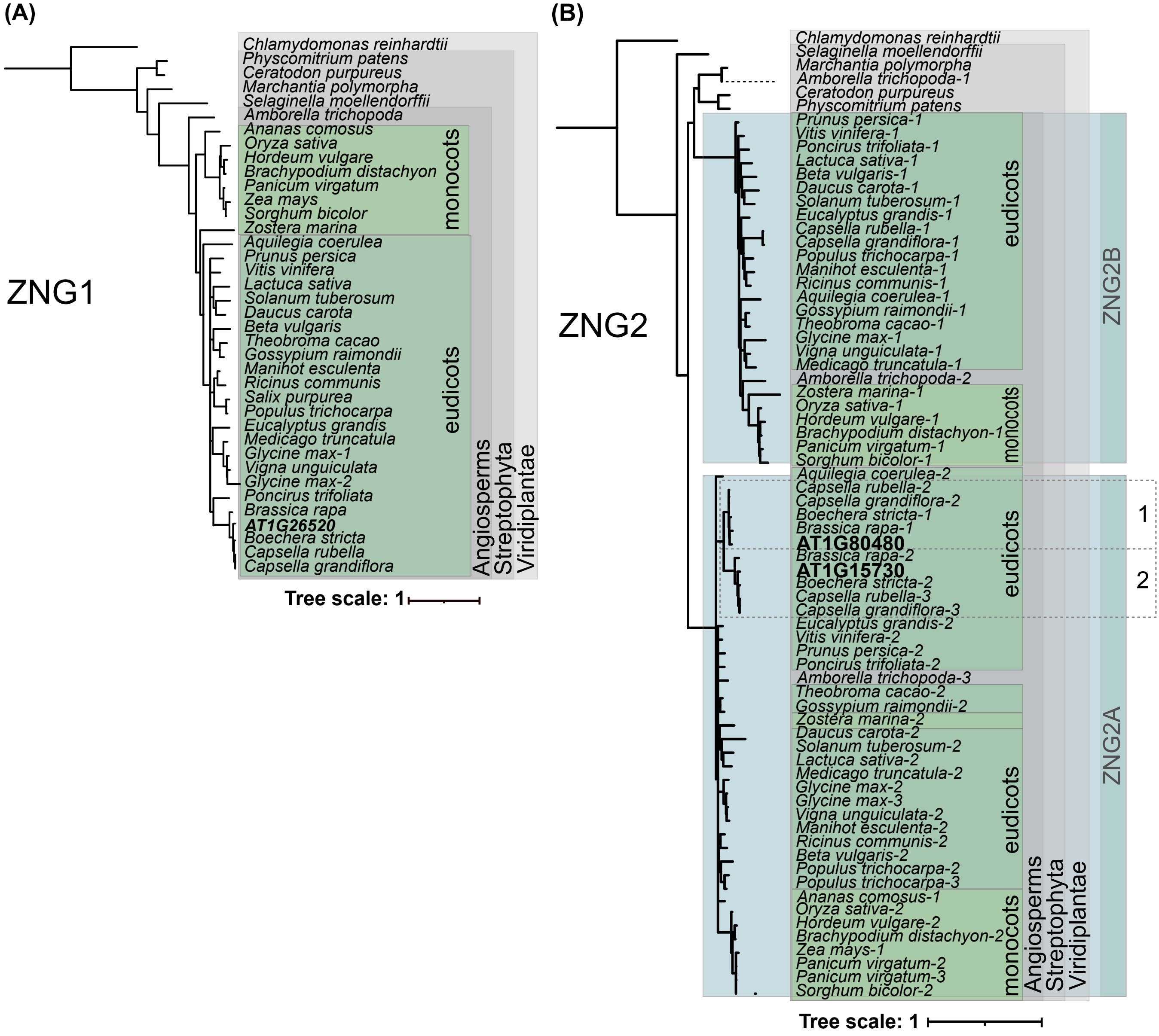

Figure S7. Phylogenetic reconstruction of plant ZNG1 and ZNG2 protein subfamilies. **(A)** Maximum likelihood phylogenetic tree of ZNG1 orthologs from selected plant genomes. Shading and labels are used to delineate different taxonomic lineages. **(B)** Maximum likelihood phylogenetic tree of ZNG2 orthologs from selected plant genomes. Green and grey shading are used to delineate different taxonomic lineages. Blue shading in used to delineate the ZNG2A and ZNG2B clades, and dotted rectangles are used to the delineate the ZNG2A paralogous groups found in Brassicaceae, represented by ZNG2A1 and ZG2A2 of *Arabidopsis thaliana*.

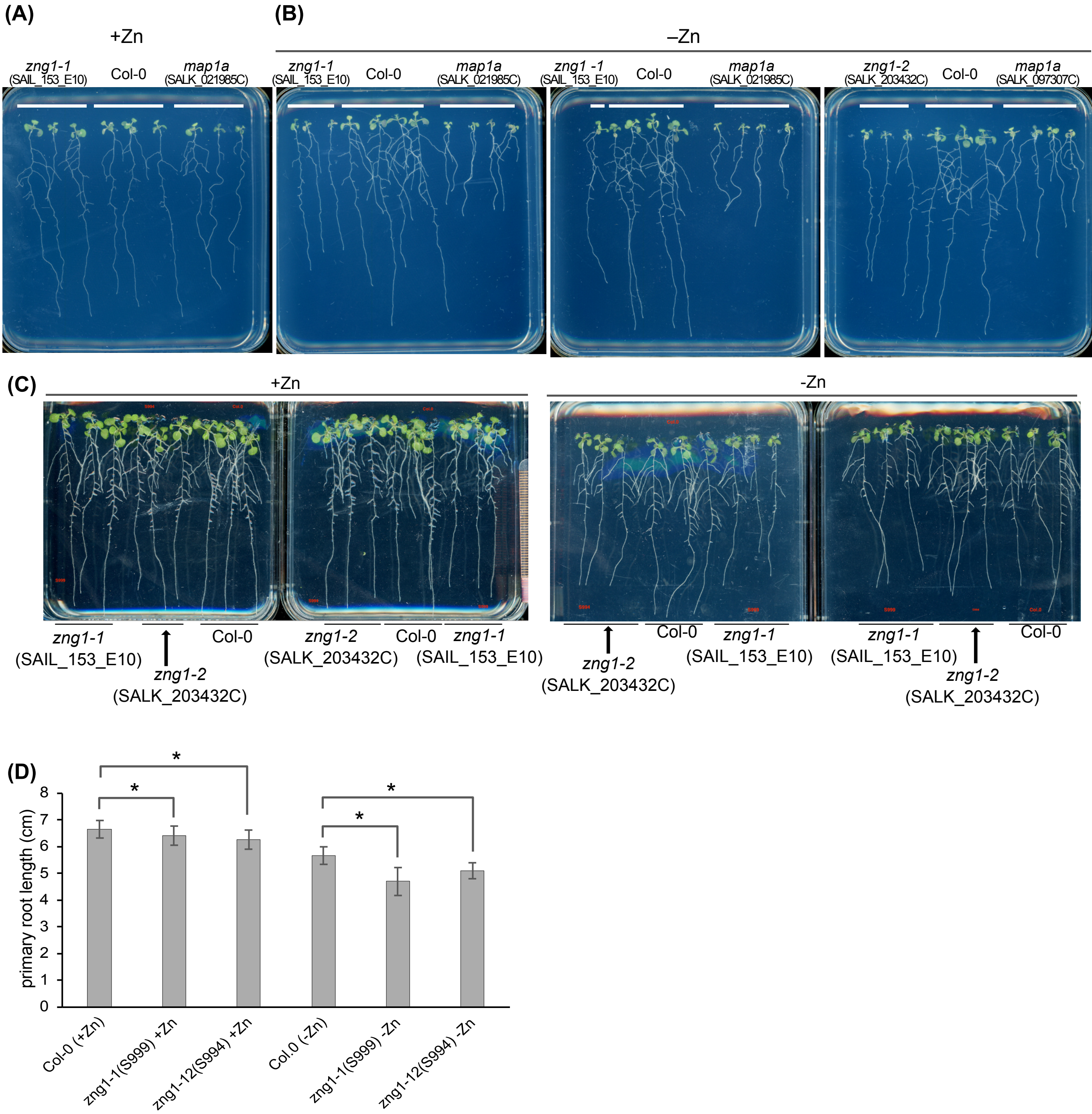

Figure S8. Impact of Zn differs with solidifying agent. **(A)** growth of *A. thaliana* lines (as indicated) on agarose-solidified medium with added Zn (10DAS). The plate contains different seedlings from the plate shown in Figure 3. **(B)** growth of *A. thaliana* lines (as indicated) on agarose-solidified medium without added Zn (10 DAS). The plates contain different seedlings from the plate shown in Figure 3, and additional mutant alleles for *zng1* and *map1a* are shown. **(C)** growth of the *zng1* mutant (SALK_203432C) on Phytagel-solidified medium with or without added Zn (as indicated) (30 DAS). **(D)** Average primary root length for each genotype and growth condition. Seedlings were grown on Phytagel-solidified medium. An average of 9 seedlings is given; three plates with three plants for each genotype per plate. Error bars represent the standard deviation. * indicates a P value < 0.05, calculated with a Student's T-test.

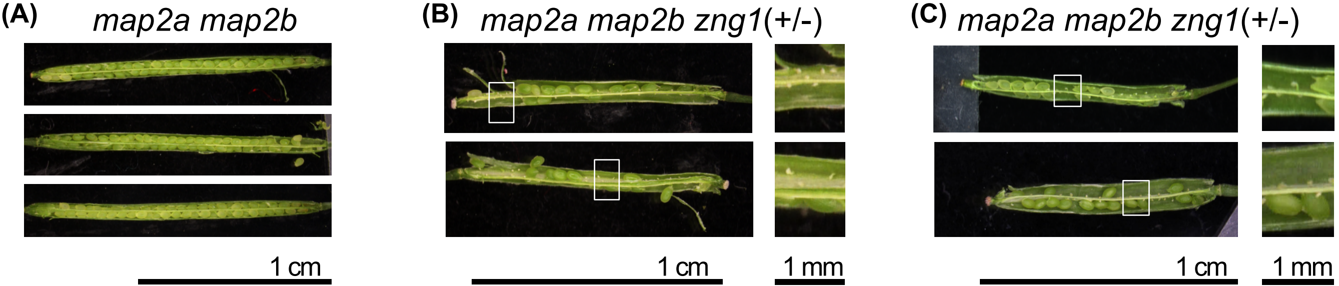

Figure S9. Aborted ovules in the siliques of *map2a map2b zng1*(+/-) plants. "+/-" is used to indicate that the *zng1* allele is heterozygous for the T-DNA insertion. **(A)** siliques from the *map2a map2b* parent. **(B)** siliques from the *map2a map2b zng1*(+/-) mutant; SALK_203432C was used as the father. **(C)** siliques from the *map2a map2b zng1*(+/-) mutant; the *zng1* mutant SAIL_153_E10 was used as the father. For **(B)** and **(C)**, the image on the right corresponds to the white box on the left.

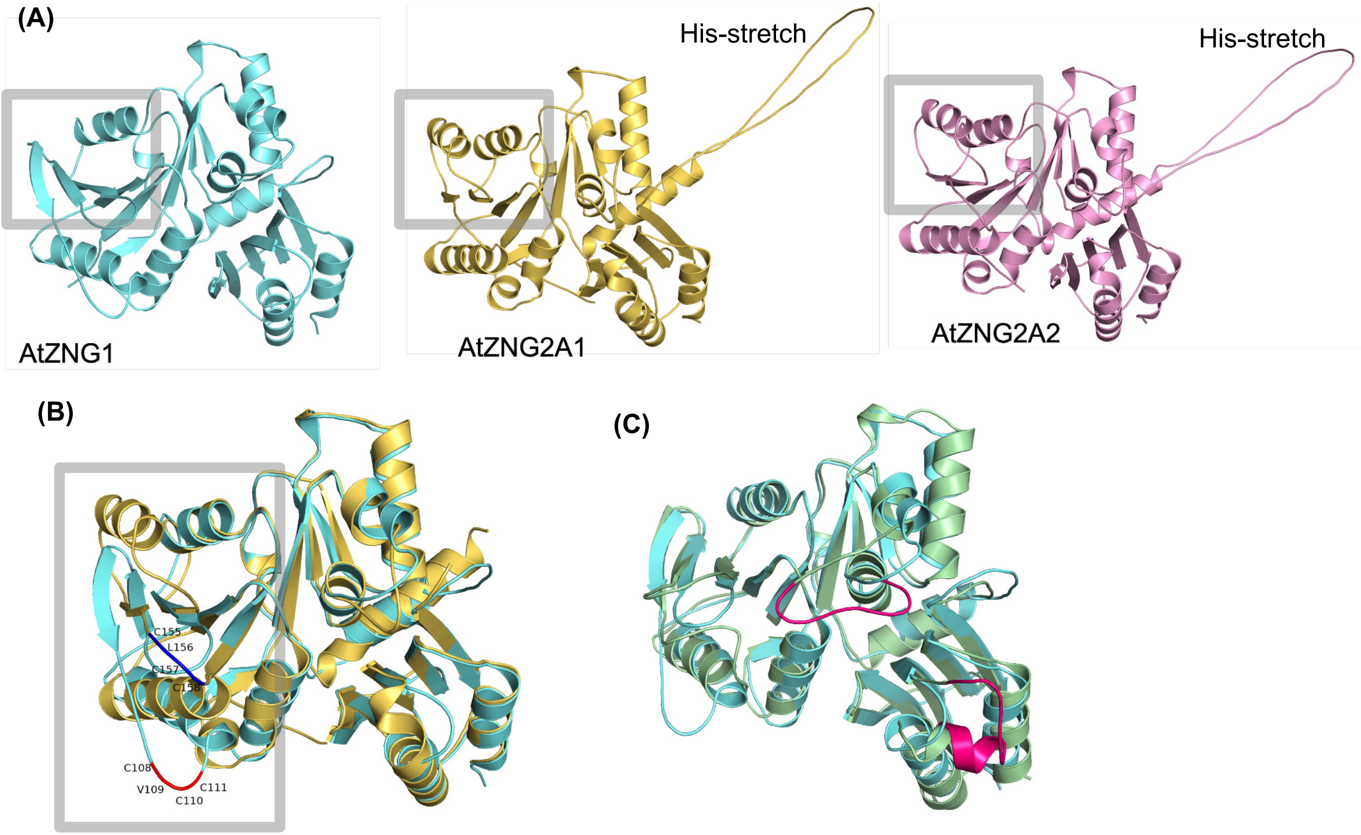

Figure S10. Predicted structures and comparisons of the *A. thaliana* ZNG proteins. **(A)** predicted structures of AtZNG1, AtZNG2A1, and AtZNG2A2. The N-terminal extension found in AtZNG1 (and other ZNG1 proteins) and the transit peptides of AtZNG2A1 and AtZNG2A2 are removed for simplicity. The major structural changes adjacent tot he CxCC motif in the AtZNG2 proteins compared to AtZNG1 are highlighted with a grey box. **(B)** Comparison of AtZNG1 (cyan) and AtZNG2A1 (gold). A grey box outlines the major structural differences that impact the position of the CxCC residues. Red is used to highlight the CxCC residues in AtZNG1 and blue is used to highlight the CxCC residues in AtZNG2A1. The N-terminal extension of AtZNG1 and transit peptide of AtZNG2A1 were removed. **(C)** Comparison of AtZNG1 (cyan) and ScZNG1 (green). Inserts found in ScZNG1 are colored pink. The N-terminal extensions were removed.

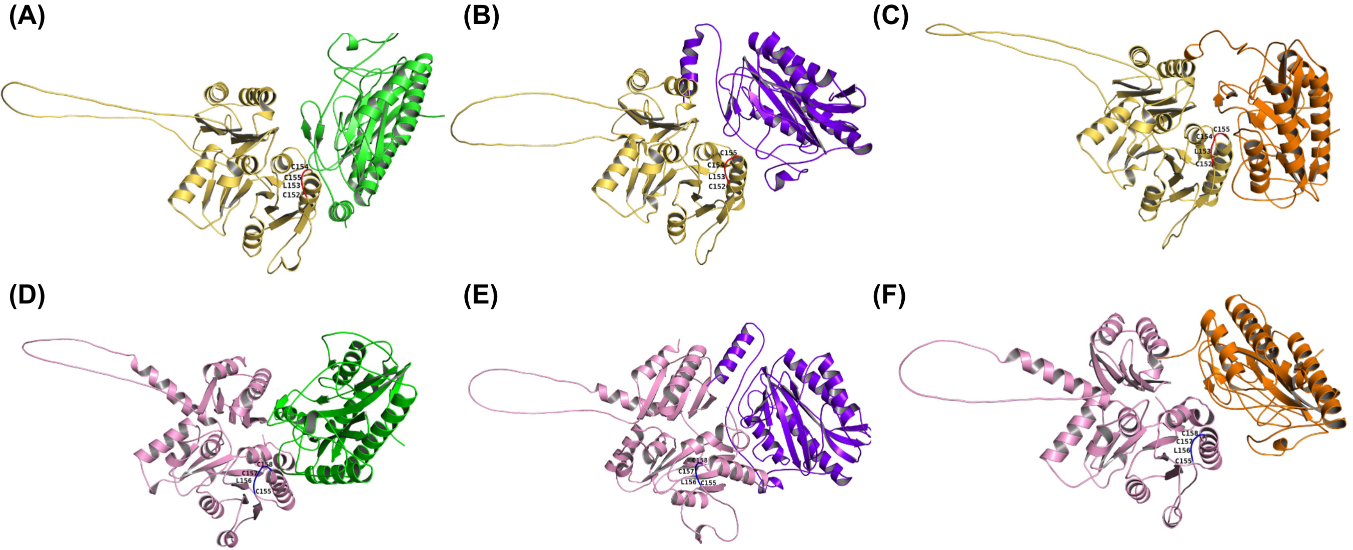

Figure S11. Predicted Alphafold complex structures. AtZNG2A1 in gold, with CxCC motif highlighted in red in complex with **(A)** AtMAP1B (green), **(B)** AtMAP1C (purple), **(C)** AtMAP1D (orange). AtZNG2A2 in pink, with CxCC motif highlighted in blue in complex with **(D)** AtMAP1B (green), **(E)** AtMAP1C (purple), **(F)** AtMAP1D. The transit peptides and unstructured N-terminal regions are not shown for image clarity [regions not shown: AtZNG2A1 (1-80), AtZNG2A2 (1-85), AtMAP1B (1-40), AtMAP1C (1-65), AtMAP1D (1-53)].
